# Extended pleurectomy decortication and chemotherapy versus chemotherapy alone for pleural mesothelioma (MARS 2): a phase 3 randomised controlled trial

**DOI:** 10.1016/S2213-2600(24)00119-X

**Published:** 2024-06

**Authors:** Eric Lim, David Waller, Kelvin Lau, Jeremy Steele, Anthony Pope, Clinton Ali, Rocco Bilancia, Manjusha Keni, Sanjay Popat, Mary O'Brien, Nadza Tokaca, Nick Maskell, Louise Stadon, Dean Fennell, Louise Nelson, John Edwards, Sara Tenconi, Laura Socci, Robert C Rintoul, Kelly Wood, Amanda Stone, Dakshinamoorthy Muthukumar, Charlotte Ingle, Paul Taylor, Laura Cove-Smith, Raffaele Califano, Yvonne Summers, Zacharias Tasigiannopoulos, Andrea Bille, Riyaz Shah, Elizabeth Fuller, Andrew Macnair, Jonathan Shamash, Talal Mansy, Richard Milton, Pek Koh, Andreea Alina Ionescu, Sarah Treece, Amy Roy, Gary Middleton, Alan Kirk, Rosie A Harris, Kate Ashton, Barbara Warnes, Emma Bridgeman, Katherine Joyce, Nicola Mills, Daisy Elliott, Nicola Farrar, Elizabeth Stokes, Vikki Hughes, Andrew G Nicholson, Chris A Rogers, MARS 2 Investigators

**Affiliations:** a Royal Brompton and Harefield Hospitals, Guy's and St Thomas' NHS Foundation Trust, London, UK; b Imperial College London, London, UK; c St Bartholomew's Hospital, London, UK; d The Clatterbridge Cancer Centre, Liverpool, UK; e Beatson West of Scotland Cancer Centre, Glasgow, UK; f Golden Jubilee National Hospital, Clydebank, UK; g University Hospitals Derby and Burton, Derby, UK; h The Royal Marsden Hospital and the Institute of Cancer Research, London, UK; i North Bristol NHS Trust, Bristol, UK; j University of Leicester, Leicester, UK; k Glenfield Hospital, Leicester, UK; l Sheffield Teaching Hospitals NHS Foundation Trust, Sheffield, UK; m Papworth Trials Unit Collaboration, Royal Papworth Hospital, Cambridge, UK; n Royal Papworth Hospital NHS Foundation Trust, Cambridge, UK; o East Sussex and North Essex NHS Foundation Trust, Colchester, UK; p Manchester University NHS Foundation Trust, Manchester, UK; q The Christie NHS Foundation Trust and Division of Cancer Sciences, University of Manchester, Manchester, UK; r Norfolk and Norwich University Hospitals NHS Foundation Trust, Norwich, UK; s Guy's and St Thomas' NHS Foundation Trust, London, UK; t Maidstone and Tunbridge Wells NHS Trust, Maidstone, UK; u South Tyneside and Sunderland NHS Foundation Trust, South Shields, UK; v Queen's Hospital, Barking Havering and Redbridge NHS Trust, Barking, UK; w South Tees Hospital NHS Foundation Trust, Middlesbrough, UK; x Leeds Teaching Hospital NHS Trust, Leeds, UK; y Royal Wolverhampton NHS Trust, Wolverhampton, UK; z Royal Gwent Hospital, Newport, UK; aa North West Anglia NHS Foundation Trust, Peterborough, UK; ab University Hospitals Plymouth NHS Trust, Plymouth, UK; ac University of Birmingham, Birmingham, UK; ad Bristol Trials Centre, Bristol Medical School, University of Bristol, Bristol, UK; ae Population Health Sciences, University of Bristol, Bristol, UK; af University of Oxford Health Economics Research Centre, Oxford, UK

## Abstract

**Background:**

Extended pleurectomy decortication for complete macroscopic resection for pleural mesothelioma has never been evaluated in a randomised trial. The aim of this study was to compare outcomes after extended pleurectomy decortication plus chemotherapy versus chemotherapy alone.

**Methods:**

MARS 2 was a phase 3, national, multicentre, open-label, parallel two-group, pragmatic, superiority randomised controlled trial conducted in the UK. The trial took place across 26 hospitals (21 recruiting only, one surgical only, and four recruiting and surgical). Following two cycles of chemotherapy, eligible participants with pleural mesothelioma were randomly assigned (1:1) to surgery and chemotherapy or chemotherapy alone using a secure web-based system. Individuals aged 16 years or older with resectable pleural mesothelioma and adequate organ and lung function were eligible for inclusion. Participants in the chemotherapy only group received two to four further cycles of chemotherapy, and participants in the surgery and chemotherapy group received pleurectomy decortication or extended pleurectomy decortication, followed by two to four further cycles of chemotherapy. It was not possible to mask allocation because the intervention was a major surgical procedure. The primary outcome was overall survival, defined as time from randomisation to death from any cause. Analyses were done on the intention-to-treat population for all outcomes, unless specified. This study is registered with ClinicalTrials.gov, NCT02040272, and is closed to new participants.

**Findings:**

Between June 19, 2015, and Jan 21, 2021, of 1030 assessed for eligibility, 335 participants were randomly assigned (169 to surgery and chemotherapy, and 166 to chemotherapy alone). 291 (87%) participants were men and 44 (13%) women, and 288 (86%) were diagnosed with epithelioid mesothelioma. At a median follow-up of 22·4 months (IQR 11·3–30·8), median survival was shorter in the surgery and chemotherapy group (19·3 months [IQR 10·0–33·7]) than in the chemotherapy alone group (24·8 months [IQR 12·6–37·4]), and the difference in restricted mean survival time at 2 years was –1·9 months (95% CI –3·4 to –0·3, p=0·019). There were 318 serious adverse events (grade ≥3) in the surgery group and 169 in the chemotherapy group (incidence rate ratio 3·6 [95% CI 2·3 to 5·5], p<0·0001), with increased incidence of cardiac (30 *vs* 12; 3·01 [1·13 to 8·02]) and respiratory (84 *vs* 34; 2·62 [1·58 to 4·33]) disorders, infection (124 *vs* 53; 2·13 [1·36 to 3·33]), and additional surgical or medical procedures (15 *vs* eight; 2·41 [1·04 to 5·57]) in the surgery group.

**Interpretation:**

Extended pleurectomy decortication was associated with worse survival to 2 years, and more serious adverse events for individuals with resectable pleural mesothelioma, compared with chemotherapy alone.

**Funding:**

National Institute for Health and Care Research (NIHR) Health Technology Assessment programme (15/188/31), Cancer Research UK Feasibility Studies Project Grant (A15895).

## Introduction

Malignant pleural mesothelioma, a cancer of the lining of the chest wall and lung associated mainly with exposure to asbestos, remains an important health issue. Despite asbestos having been banned in Europe since 2005 and controlled in the USA for more than 40 years, the incidence rate per 100 000 people has been reported as 0·9 for men and 0·3 for women in the USA, and as 1·7 for men and 0·4 for women in Europe.^1^ With an average reported lifespan from diagnosis of 9–12 months,^1^ malignant pleural mesothelioma is one of the most aggressive cancers, with platinum and pemetrexed chemotherapy^2^ the mainstay systemic treatment for early-stage resectable disease despite its modest effect on survival. With decades of little progress in systemic treatment for malignant pleural mesothelioma, surgery to remove all visible disease (macroscopic resection or cytoreduction) is widely offered and uniformly recommended in American guidelines,^3^, ^4^ European guidelines,^5^, ^6^ and international consensus statements^7^ as the principal option to improve survival.

**Research in context**

**Evidence before this study**

We conducted a review of the literature on PubMed from Jan 1, 1966, to Sept 1, 2017, restricted to randomised trials of extended pleurectomy decortication for malignant pleural mesothelioma, and none were identified. Data from cases series have been the principal evidence base for surgery worldwide but, in the absence of high-quality controlled or comparable data, it is not possible to draw robust inferences on the effect of extended pleurectomy decortication surgery on survival or quality of life.

**Added value of this study**

The MARS 2 multicentre randomised controlled trial sought to evaluate the effect of extended pleurectomy decortication surgery on survival and quality of life, and provide randomised, controlled evidence to guide the management of individuals with pleural mesothelioma. Extended pleurectomy decortication and chemotherapy did not improve overall survival compared with chemotherapy alone; the median survival from randomisation was shorter at 19·3 months in the chemotherapy plus surgery group compared with 24·8 months in the chemotherapy alone group. Surgery was associated with a 3·6 times greater number of serious adverse events compared with chemotherapy alone. Differences in patient-reported quality of life favoured the chemotherapy alone group. Surgery was associated with significantly fewer quality-adjusted life years and higher costs than chemotherapy alone; at all willingness-to-pay thresholds, surgery was not considered cost-effective.

**Implications of all the available evidence**

All randomised trials published to date have reported higher risk of death with cytoreductive surgery. Reconsideration of the concept of surgically resectable disease has the potential to improve survival by facilitating access to effective systemic treatments currently denied to individuals with resectable disease.

There are two main surgical techniques^8^ for macroscopic resection (maximal cytoreduction). The first is extrapleural pneumonectomy, which consists of en bloc resection of the parietal and visceral pleura with the ipsilateral lung, pericardium, and diaphragm. Extrapleural pneumonectomy was evaluated in a UK-based randomised controlled trial (MARS),^9^ reporting a higher risk of death and poorer quality of life. Therefore, the second technique, a less morbid and less extensive operation, extended pleurectomy decortication—which consists of parietal and visceral pleurectomy to remove all gross tumour and, where required, the additional resection of the diaphragm or pericardium, but spares the underlying lung—is currently offered as the principal surgical procedure worldwide, despite the absence of any randomised evidence to support clinical effectiveness.

The MARS 2 multicentre randomised trial sought to assess the clinical effectiveness, safety, quality of life, and cost-effectiveness of extended pleurectomy decortication and chemotherapy compared with chemotherapy alone in individuals with resectable pleural mesothelioma.

## Methods

### Study design

Full details of the MARS 2 trial design have been published^10^ and additional details are provided in the appendix (pp 9–12). MARS 2 was a national, multicentre, open-label, parallel two-group, pragmatic, superiority randomised controlled trial conducted in 26 hospitals (21 recruiting only, one surgical only, and four recruiting and surgical). The research protocol was approved by Camberwell St Giles Research Ethics Committee (13/LO/1481). The trial was overseen by an independent trial steering committee and an independent data and safety monitoring committee (appendix pp 8–9). This study is registered with ClinicalTrials.gov, NCT02040272.

### Participants

Participants were recruited between June 19, 2015, and Jan 21, 2021. Trial recruitment was augmented by the QuinteT Recruitment Intervention.^11^ Individuals with cytologically or histologically confirmed epithelioid, sarcomatoid, or biphasic mesothelioma disease confined to one hemithorax, as confirmed by CT assessment, and deemed to be surgically resectable by a surgeon at a MARS 2 surgical site (to ensure centralisation and quality control) and regional specialist mesothelioma multidisciplinary team, were eligible for participation. Participants had to be 16 years of age or older and have adequate organ function and lung function to be included (full inclusion and exclusion criteria are described in the appendix, p 9). Key exclusion criteria were severe comorbidities that would preclude surgery. All participants provided written informed consent.

### Randomisation and masking

Following two cycles of chemotherapy, eligible participants were randomly assigned (1:1) to surgery and chemotherapy or chemotherapy alone using a secure web-based system (Sealed Envelope). Allocation was stratified by site and minimised by age, performance status, and cell type. It was not possible to mask the allocation as the intervention was a major surgical procedure.

### Procedures

Consenting participants underwent two cycles (3 weeks per cycle) of standard-of-care chemotherapy (eg, platinum and pemetrexed) followed by a repeat CT chest scan using a contrast and recommended pleural enhancement protocol. Images were reviewed by the multidisciplinary team and surgeon at a MARS 2 surgical site to confirm ongoing eligibility (ie, that any progression was still within surgically resectable limits).

Participants in the chemotherapy only group (comparator group) received two to four further cycles of chemotherapy, according to local treatment policies. Participants in the surgery and chemotherapy group (experimental group) received pleurectomy decortication (parietal and visceral pleurectomy to remove all gross tumour) or extended pleurectomy decortication (parietal and visceral pleurectomy to remove all gross tumour with resection of the diaphragm or pericardium), in accordance with international consensus, followed by two to four further cycles of chemotherapy, according to local treatment policies.^8^ After participants had completed their trial treatment, no restrictions were placed on participation in further clinical trials or approved systemic treatments.

All 26 treatment sites were NHS Trusts with specialist mesothelioma services and a dedicated mesothelioma multidisciplinary team. The five surgical sites (four established UK centres for mesothelioma surgery and, by design,^10^ one non-expert surgical centre) had the additional requirement of at least two mesothelioma surgeons who had undergone peer-reviewed observation of (extended) pleurectomy decortication surgery as part of the MARS 2 trial quality assurance protocol.^10^

Participating surgeons in the full trial had to be accredited by a quality assurance protocol that stated: a minimum of five (extended) pleurectomy decortication surgeries undertaken before joining the trial; observing the procedure being undertaken at an established MARS 2 surgical site; having their first surgery undertaken in the trial observed by a surgeon from the pilot phase; and having one randomly selected MARS 2 operation between procedures 5 and 10 observed by a surgeon from the pilot phase to ensure fidelity. These minimum entry requirements were principally intended for surgeons at the non-expert centre; the vast majority of procedures were undertaken by surgeons in expert centres with career case volumes far in excess of the minimum stipulated requirement.

### Outcomes

The primary outcome was overall survival (defined as time from randomisation to death from any cause). Prespecified secondary outcomes were investigator-assessed progression-free survival as per local imaging and follow-up, serious adverse health events graded using Common Terminology Criteria for Adverse Events (version 4.0), health-related quality of life (HRQoL) assessed using the European Organisation for Research and Treatment of Cancer (EORTC) core health-related quality of life questionnaire (QLQ-C30) and five-level EQ-5D self-report questionnaires, and health-care resource use on intervals specified by the protocol up to 24 months from randomisation.

### Statistical analysis

We hypothesised that overall survival for participants undergoing (extended) pleurectomy decortication would improve by 30% (hazard ratio [HR] 0·70), the minimum clinically important difference chosen by participants and clinicians given the magnitude and risks associated with the operation. The possibility of worse survival was also considered; therefore, a two-tailed test was agreed. The sample size was set at 328 participants (164 per group) to provide 80% power to detect an HR of 0·70 at 5% statistical significance, assuming a median survival of 16·8 months in the chemotherapy alone group and allowing for up to 10% crossover (appendix p 10).

Analyses were performed on an intention-to-treat basis for all outcomes unless otherwise stated. Survival outcomes, including progression-free survival, were compared using Cox proportional hazards regression if the proportional hazards assumption held. If the assumption was violated, an analysis of the restricted mean survival time to 2 years, the minimum follow-up time for all participants, was used. Participants who were alive at their last known follow-up were censored from that point. The proportional hazards assumption was assessed graphically and statistically (appendix p 10). Safety outcomes of total count and occurrence of specific events were analysed using mixed-effects generalised linear models accounting for follow-up time, and treatment effects are presented as incidence rate ratios (IRRs). Longitudinal HRQoL was analysed using joint longitudinal survival models,^12^ with results presented as mean differences (appendix p 11). Methods for handling missing data are described in the appendix (p 11). Adjustments for multiplicity in HRQoL and safety outcomes were performed using the Benjamini–Hochberg method.^13^ For survival outcomes with non-proportional hazards, we also performed Cox regression, splitting the survival time into epochs defined by point(s) where the Kaplan–Meier curves crossed. A prespecified subgroup analysis compared the primary outcome by mesothelioma cell type (epithelioid versus non-epithelioid), and sensitivity analyses explored the effect of the number of first-line chemotherapy cycles and additional treatments received (this accounted for treatments received on the CONFIRM trial, which required unblinding,^14^ for which immunotherapy was the investigational product). All sensitivity and exploratory analysis are detailed in the appendix (pp 11–12). Instrumental variable methods using randomised group as the instrument were used to assess the effect of non-adherence with the intervention. The analyses assessing the effect of non-adherence were implemented using two-stage least-squares instrumental variable methods. In the first stage, treatment received (exposure) was regressed on randomised treatment group (instrument) and predicted probabilities were obtained. In the second stage, the outcome was regressed on the predicted exposure. All other covariates remained the same as in the primary analyses. The effects of surgical site expertise and of surgeon were explored (appendix p 11). We performed post-hoc analyses to examine the primary outcome for: PET-CT versus no PET-CT; year randomised; and the ideal selection cohort (T1–2, N0, epithelioid-only disease).

All statistical analyses were performed using Stata version 17.0.

### Economic evaluation

A within-trial economic evaluation was undertaken from an NHS and personal social services perspective, with a time horizon from time of consent to 24 months after randomisation. The primary outcome was quality-adjusted life years, a measure combining length and quality of life, estimated using the five-level EQ-5D. Data were collected on all relevant health and social care inputs at each follow-up timepoint, and valued in 2021–22 pounds sterling using national data sources (based on actual costs rather than on charges) and converted to US dollars (£1=$1·37; appendix p 32).

### Role of the funding source

The funder had no role in data collection, analysis, interpretation, writing of the manuscript, or the decision to submit for publication.

## Results

**Figure 1:**
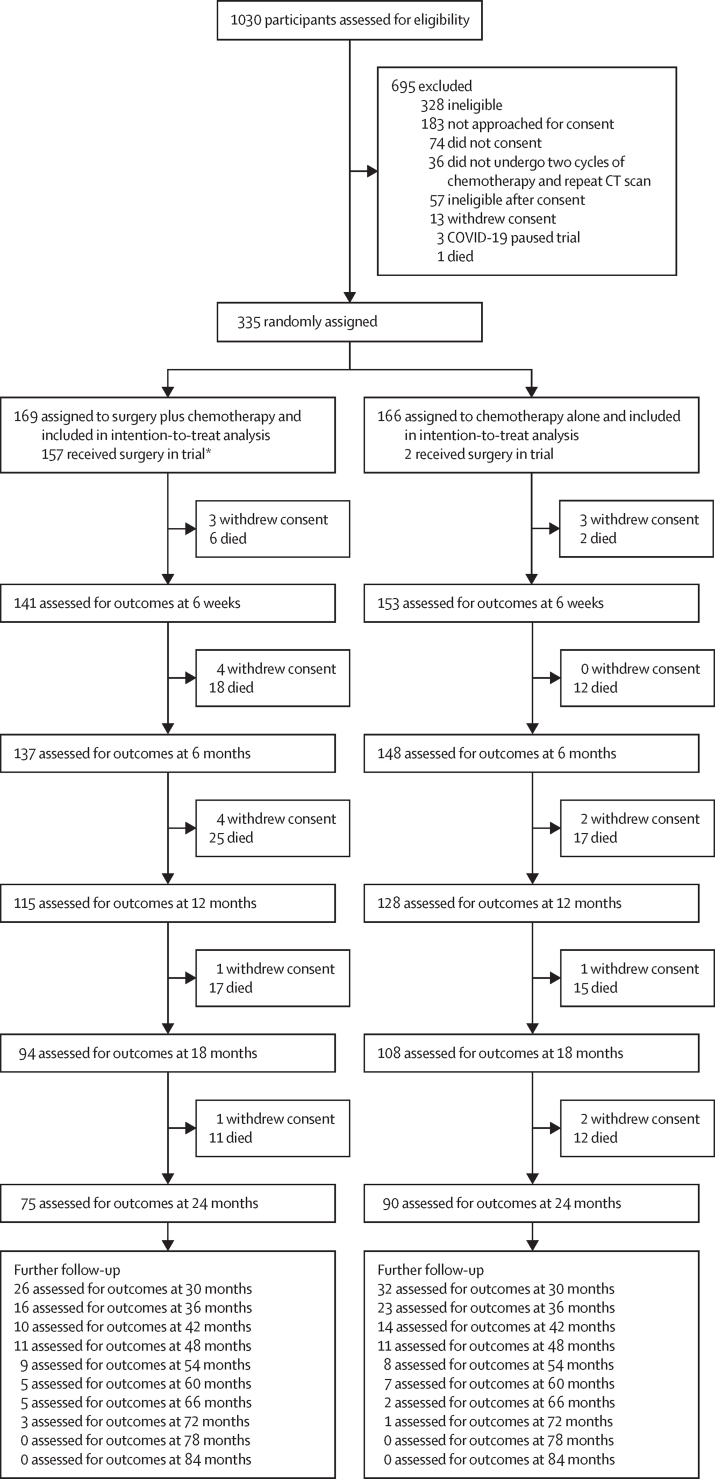
Trial profile Reasons why participants did not complete the initial two cycles of chemotherapy or have a repeat CT are provided in the appendix (p 24). Of the 392 participants who underwent a repeat CT scan after the initial two courses of chemotherapy, 35 had disease progression beyond surgically resectable limits and were excluded due to ineligibility. A further 33 participants had evidence of disease progression but were still deemed to be surgically resectable; 28 of these 33 participants were randomly assigned. *One participant assigned to the surgery group withdrew and received surgery privately, so no surgical data were available for this participant. One participant in the surgery group and two participants in the no-surgery group withdrew from completing quality-of-life questionnaires but continued with clinical follow-up and so are not included as withdrawals, as they were assessed for outcomes following the withdrawal.

Between June 19, 2015, and Jan 21, 2021, 1030 participants from 26 sites were screened for eligibility, with 392 participants undergoing a repeat staging CT after two initial cycles of chemotherapy. Of these 392 participants, 35 were excluded due to progression beyond surgically resectable limits and 33 included with progression within surgically resectable limits, of whom 28 proceeded to randomisation (8% of the total randomisation cohort). Three patients withdrew as they no longer wanted surgery, one had pulmonary embolisms and deteriorating performance status, and one had clear evidence of seventh rib destruction so was withdrawn because their quality of life would have been severely affected by surgery. In total, 335 participants were randomly assigned (169 [50%] to surgery and chemotherapy and 166 [50%] to chemotherapy alone; figure 1). Baseline characteristics were well balanced between the two groups (table, appendix pp 22–23), with 288 (86%) participants diagnosed with epithelioid mesothelioma and a minority with biphasic (29 [9%]) or sarcomatoid (11 [3%]) disease.

**Table:** Baseline characteristics and systemic treatments

		**Chemotherapy with surgery (N=169)**	**Chemotherapy with no surgery (N=166)**
Age, years		69 (7)	69 (7)
Sex			
	Male	152/169 (90%)	139/166 (84%)
	Female	17/169 (10%)	27/166 (16%)
ECOG status (baseline)			
	0	82/169 (49%)	69/166 (42%)
	1	87/169 (52%)	97/166 (58%)
CRP, mg/L*		16 (7–57)	10 (5–44)
White cell count, ×10^9^/L†		8 (7–10)	8 (7–10)
Platelets, ×10^9^/L†		315 (265–405)	314 (252–398)
Albumin, g/dL‡		3·9 (3·6–4·3)	4·1 (3·6–4·3)
Haemoglobin, g/dL†		14 (13–15)	14 (13–15)
Cell type (randomisation)			
	Epithelioid only	145/169 (86%)	142/166 (86%)
	Other	24/169 (14%)	24/166 (15%)
Histological type or subtype§			
	Epithelioid mesothelioma	145/169 (86%)	143/166 (86%)
	Sarcomatoid mesothelioma	8/169 (5%)	3/166 (2%)
	Biphasic mesothelioma	13/169 (8%)	16/166 (10%)
	Other (desmoplastic or not specified) mesothelioma	2/169 (1%)	3/166 (2%)
	Unable to classify	1/169 (1%)	1/166 (1%)
cT			
	T1	75/169 (44%)	81/166 (49%)
	T2	36/169 (21%)	36/166 (22%)
	T2: involvement of diaphragmatic muscle	9/36 (25%)	21/36 (58%)
	T2: extension of tumour into underlying pulmonary parenchyma	30/36 (83%)	18/36 (50%)
	T3	58/169 (34%)	49/166 (30%)
	T3: involvement of endothoracic fascia	20/58 (35%)	17/50 (34%)
	T3: extension into mediastinal fat	30/58 (52%)	30/50 (60%)
	T3: solitary, completely resectable focus of tumour extending into soft tissues of chest wall	18/58 (31%)	14/50 (28%)
	T3: non-transmural involvement of pericardium	16/58 (28%)	10/50 (20%)
cN			
	N0	122/169 (72%)	119/166 (72%)
	N1	34/169 (20%)	36/166 (22%)
	N2	13/169 (8%)	11/166 (7%)
cM			
	M0	163/169 (96%)	162/166 (98%)
	M1	6/169 (4%)	4/166 (2%)
Smoking status (ever)		97/168 (58%)	94/166 (57%)
FEV_1_, % predicted¶		75% (19)	75% (17)
FVC, % predicted‖		78% (18)	79% (22)
TLCO, % predicted**		74% (19)	78% (21)
First-line chemotherapy cycles††			
	Completed 2 cycles	169/169 (100%)	166/166 (100%)
	Completed 3 cycles	101/169 (60%)	154/166 (93%)
	Completed 4 cycles	96/169 (57%)	147/166 (89%)
	Completed 5 cycles	76/169 (45%)	111/166 (67%)
	Completed 6 cycles	66/169 (39%)	93/166 (56%)
Any additional treatment reported during trial participation		90/169 (53%)	115/166 (69%)
	Immunotherapy or other treatment known to improve overall survival‡‡	37/169 (22%)	64/166 (39%)
	Additional chemotherapy	35/169 (21%)	65/166 (39%)
	Radiotherapy	32/169 (19%)	30/166 (18%)
	Further surgery	4/169 (2%)	6/166 (4%)
	Other systemic treatment	10/169 (6%)	19/166 (11%)

Data are median (IQR), mean (SD), or n/N (%). CRP=C-reactive protein. ECOG=Eastern Cooperative Oncology Group. FVC=forced vital capacity. TLCO=transfer factor of the lung for carbon monoxide.

* 251 patients with missing data (131 with surgery, 120 with no surgery).

† Two patients with missing data (both with no surgery).

‡ Seven patients with missing data (four with surgery, three with no surgery).

§ Histological type or subtype presented is histological type at baseline, prior to randomisation. Any deemed unable to classify at baseline have been updated following the histological review, where possible (appendix p 23).

¶ 84 patients with missing data (38 with surgery, 46 with no surgery).

‖ 133 patients with missing data (67 with surgery, 66 with no surgery).

** 159 patients with missing data (84 with surgery, 75 with no surgery).

†† Reasons for not starting cycle 3 are presented in the appendix (p 25).

‡‡ Includes pembrolizumab, nivolumab, and bevacizumab.

Two (1%) of 166 participants assigned to chemotherapy alone crossed over to chemotherapy with surgery, 13 (8%) of 169 assigned to chemotherapy with surgery crossed over to chemotherapy alone, and 24 participants withdrew after randomisation. Primary outcome data were available for all randomly assigned participants except one (figure 1). The analysis population included all randomly assigned participants. Reasons for ineligibility, non-consent, protocol deviations, and withdrawals after randomisation are presented in the appendix (pp 24–25).

For the participants who received surgery, the operations were undertaken in recognised centres of expertise for mesothelioma surgery and, by design, one non-expert centre.^10^ The International Association for the Study of Lung Cancer consensus statement for surgery for mesothelioma^15^ defined low volume as fewer than five procedures per year, and the average case volumes for radical surgery for mesothelioma were 8·6, 14·3, 27·6, and 36·5 cases per year (by rank) in the expert mesothelioma surgery centres and 3·4 cases per year in the non-expert centre (which performed 11 [7%] of 157 total operations) during the MARS 2 trial. The reported surgical volumes were reached despite 166 otherwise eligible surgical patients being randomly assigned to chemotherapy (as part of MARS 2) and reduced operations countrywide due to the effects of the COVID-19 pandemic. The majority received either extended pleurectomy decortication (139 [89%] of 157) or pleurectomy decortication (13 [8%] of 157; appendix pp 26–27). The median length of hospital stay was 13 days (IQR 12–14). The in-hospital mortality and 30-day mortality for those who underwent surgery were both six (4%) of 157, and the 90-day mortality was 14 (9%). The completeness of resection was microscopic residual disease (R1) in 127 (81%) and complete microscopic clearance (R0) in five (3%). Fewer participants (66 [39%] of 169) randomly assigned to surgery received six cycles of chemotherapy and additional treatments such as immunotherapy compared with those randomly assigned to chemotherapy alone (93 [56%] of 166; table).

**Figure 2:**
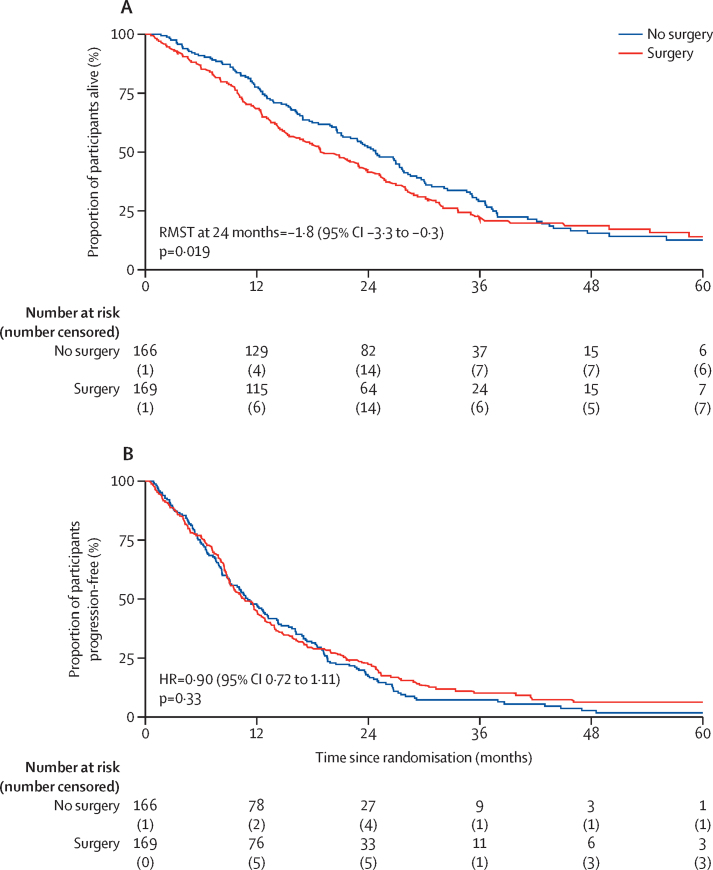
(A) Overall survival and (B) progression-free survival (A) Median survival: surgery 19·3 months (IQR 10·0–33·7), no surgery 24·8 months (12·6–37·4). Survival probabilities at 24 months: surgery 0·41 (95% CI 0·34–0·49), no surgery 0·52 (0·44–0·59). (B) Median progression-free survival: surgery 10·6 months (IQR 6·3–21·6), no surgery 11·0 months (5·9–19·6). Survival probabilities at 24 months: surgery 0·22 (95% CI 0·16–0·29), no surgery 0·17 (0·12–0·24). HR=hazard ratio. RMST=restricted mean survival time.

At a median follow-up of 22·4 months (IQR 11·3–30·8), the median survival of participants assigned to surgery and chemotherapy was 19·3 months (10·0–33·7) compared with 24·8 months (12·6–37·4) for participants assigned to chemotherapy alone (difference in restricted mean survival time to 24 months of –1·9 months [95% CI –3·4 to –0·3, p=0·019] in favour of chemotherapy alone; figure 2A). In the first 42 months (the point where the Kaplan–Meier curves intersected), the HR for participants randomly assigned to surgery and chemotherapy versus chemotherapy alone was 1·28 (95% CI 1·02 to 1·60, p=0·032), indicating a 28% increase in the risk of death in the surgery group. The number of participants (n=30) in follow-up beyond 42 months was too few for meaningful comparative analysis. The survival rate was 14% (95% CI 8 to 22) in the surgery and chemotherapy group and 13% (7 to 20) in the chemotherapy alone group at 5 years, with most deaths due to disease progression (appendix p 27).

There was no difference in progression-free survival (figure 2B, appendix p 27), with a median of 10·6 months (IQR 6·3–21·6) in the surgery and chemotherapy group and 11·0 months (5·9–19·6) in the chemotherapy alone group (HR 0·90 [95% CI 0·72–1·11], p=0·33).

There were 318 serious adverse events (grade ≥3) in the surgery group and 169 in the chemotherapy alone group. There was a median of one (IQR 0–3) serious adverse event per person in the surgery group and zero (0–2) in the chemotherapy alone group (IRR 3·6 [95% CI 2·3–5·5], p<0·0001), defined as Common Terminology Criteria for Adverse Events grade 3 and above, and this centred on an increased incidence of cardiac (30 *vs* 12 events; 3·01 [1·13–8·02]) and respiratory (84 *vs* 34 events; 2·62 [1·58–4·33]) disorders, infection (124 *vs* 53 events; 2·13 [1·36–3·33]), and additional surgical or medical procedures (15 *vs* eight events; 2·41 [1·04–5·57]) in the surgery group (appendix pp 13, 27–30).

**Figure 3:**
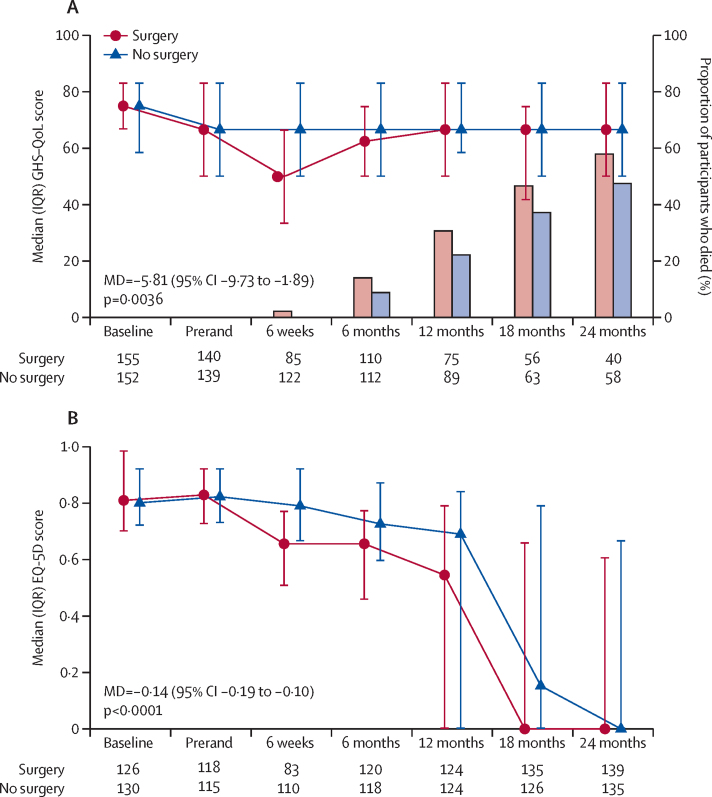
(A) GHS–QoL scores and (B) EQ-5D utility scores over time in survivors QLQ-C30 GHS–QoL scores range from 0 to 100; higher scores indicate better health. For EQ-5D, scores range from –0·594 to 1; higher scores indicate better quality of life, and death has a score of zero. GHS–QoL=global health status–quality of life. MD=mean difference. Prerand=prerandomisation. QLQ-C30=European Organisation for Research and Treatment of Cancer core health-related quality of life questionnaire.

All statistically significant differences in EORTC HRQoL scales favoured chemotherapy alone. For global health and social functioning, the mean difference between groups did not vary with follow-up time (global health –5·51 [95% CI –9·73 to –1·89] and social functioning –10·87 [–16·07 to –5·66]; figure 3A, appendix p 14). For physical and role functioning, the mean difference between groups reduced over time, with peak mean differences at 6 weeks of –11·46 (–15·39 to –7·52) for physical functioning and –15·77 (–22·03 to –9·50) for role functioning (appendix pp 14–15, 31). Positive symptom scores were also worse for participants in the surgery group with pain (peak mean difference at 6 weeks 25·98 [19·64 to 32·31]), fatigue (peak mean difference at 6 weeks 10·78 [4·65 to 16·92]), dyspnoea (peak odds ratio [OR] at 6 weeks 4·28 [2·42 to 7·55]), insomnia (peak OR at 6 weeks 2·15 [10·8 to 4·28]), loss of appetite (peak OR at 6 weeks 2·93 [1·30 to 6·60]), and financial difficulties (peak OR at 6 weeks 10·61 [2·99 to 37·61]; appendix pp 18–20, 31–32). EORTC HRQoL data could only be collected from participants who were alive at the time of scheduled data collection. To guard against a healthy cohort effect, EQ-5D utility scores are presented revealing the effect of death (figure 3B). Subscale EORTC QLQ-C30 scores are summarised in the appendix (pp 14–20).

**Figure 4:**
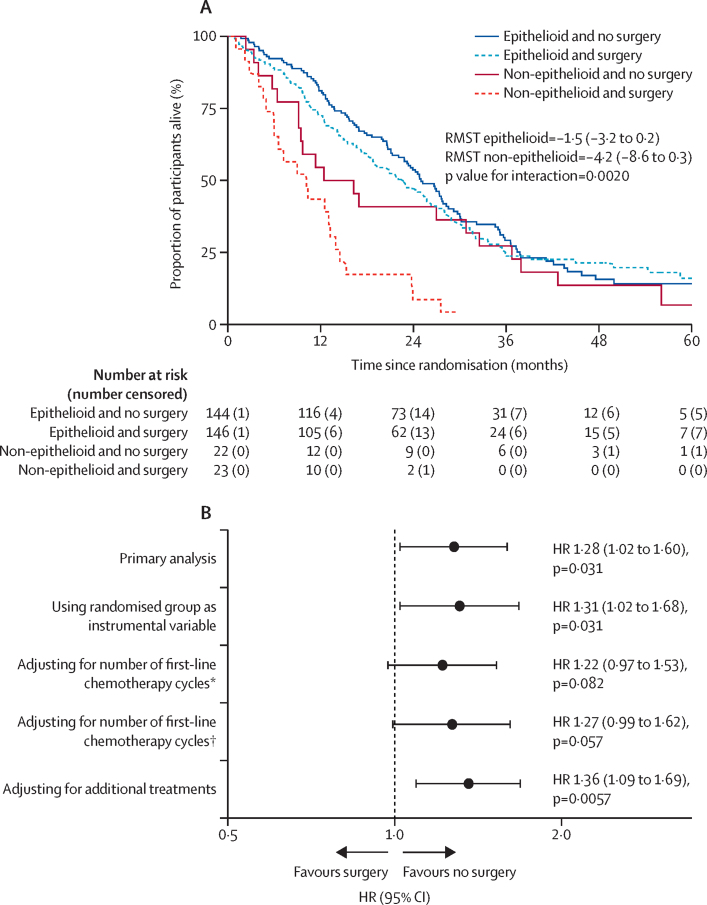
(A) Survival by histological subtype and (B) primary outcome sensitivity and exploratory analyses Estimates are provided for the period 0–42 months. Instrumental variable analysis accounts for crossovers. HR=hazard ratio. RMST=restricted mean survival time, months (95% CI). *Analysis using 4 × average half-life of chemotherapy drugs received to determine when time-varying covariate is switched off (=0). †Analysis turning time-varying covariate off (=0) 21 days after the end of chemotherapy (appendix pp 11–12).

The effect of surgery and chemotherapy versus chemotherapy alone on overall survival differed between participants with pure epithelioid histology (HR 1·12 [95% CI 0·86–1·47]) and all other subtypes (2·66 [1·22–5·81], p=0·049; figure 4A). There was no statistically significant difference in survival between expert and non-expert surgical sites (p=0·51), nor between individual surgeons (p=0·38). The estimates of HR for death after adjusting for the number of cycles of first-line chemotherapy, additional immunotherapy, and participation in clinical trials (appendix p 32) and when accounting for crossovers were consistent with the primary analysis (figure 4B). Differences in progression-free survival and number of serious adverse events when accounting for crossovers were also consistent with the primary analyses (appendix pp 13, 32).

Mean quality-adjusted life years for participants randomly assigned to surgery and chemotherapy were 1·02 (95% CI 0·94 to 1·10) compared with 1·21 (1·13 to 1·29) for participants assigned to chemotherapy alone, with a mean difference of –0·19 (95% CI –0·30 to –0·08). The mean total costs of care were £30 436 (US$41 871) in the surgery and chemotherapy group and £15 805 ($21 743) in the chemotherapy alone group, with a mean difference of £14 631 ($20 128) and 95% CI of £11 279 to £17 983 ($15 517 to $24 740), as detailed in the appendix (p 33). This difference is driven by the substantial costs of surgery.

## Discussion

In the MARS 2 trial, participants with resectable pleural mesothelioma who received (extended) pleurectomy decortication and chemotherapy had significantly worse survival, more serious adverse events, poorer quality of life, and higher costs than those who received chemotherapy alone.

Pleurectomy decortication for the treatment of mesothelioma was reported in the literature as early as 1976^16^ and is currently the most commonly performed surgical procedure for maximum cytoreduction in individuals with non-widespread, early-stage disease.^15^ Despite uniform international consensus and recommendations for (extended) pleurectomy decortication in all the major guidelines, its efficacy has never been evaluated in a randomised trial. Until now, presumed efficacy has been justified by outcomes of surgical case series^17^ (influenced by selection bias) or comparative efficacy against extrapleural pneumonectomy^18^ (a more morbid operation). The MARS 2 cohort randomly assigned to chemotherapy alone had a median survival of 24·8 months, higher than that of well cited surgical cohorts for (extended) pleurectomy decortication at 20·7 months^17^ but lower than the best reported median surgical survival of 35·6 months.^19^ Ultimately it is not possible to compare between surgical series, due to differences in case selection, numbers, and follow-up time, underscoring the importance of a randomised design to evaluate the outcomes of surgical procedures against a similar comparative non-surgical control group.

The survival profile of the MARS 2 cohort was not proportional (the curves meet at approximately 42 months); therefore, restricted mean survival time was specified in our statistical analysis plan to compare survival time between the two groups at 2 years, which was anticipated to reflect the optimal timepoint for the effect of surgery. We also modelled survival in two separate timeframes (ensuring validity of the proportional hazards assumption) to facilitate clinical interpretation of the magnitude of early hazard of death. At face value, we would consider any statistically significant difference that reduces survival to be harmful (counter to the purpose of the intervention), but to interpret the argument symmetrically, we could consider 30% reduction in survival as harmful (recognising that most authors provide less magnitude or accept higher p values to indicate harm). The HR was 1·28, just short of a symmetrical 30% value; as such, we simply conclude that surgery was associated with a higher risk of death.

In addition to poorer survival, participants in the surgery group had a 3·6-fold increase in serious adverse events compared with the chemotherapy alone group. Although quality-of-life scores appear to come together after 6 months (figure 3A), the interpretation of quality-of-life measures associated with recovery is biased in favour of an intervention with a substantial number of deaths, as it is dependent on being alive and sufficiently well to report (participants too unwell would not be able to report and those who died would be censored). Only when quality of life and death are jointly taken into account (using the EQ-5D utility score; figure 3B) can we appreciate the effect of surgery on participants with significantly worse utility throughout follow-up. Surgery is associated with both statistically significantly fewer quality-adjusted life years and higher costs than chemotherapy alone; hence, surgery is clearly not cost-effective or a good use of resources. In contrast, chemotherapy alone is considered cost-effective at any willingness-to-pay threshold, and there is negligible uncertainty around this finding.

As systemic treatments continue to improve survival outcomes in pleural mesothelioma, it is imperative to estimate the efficacy of surgery in resectable disease. This is because current trials of systemic therapies are often based on the concept of unresectable disease as a prerequisite for trial entry,^20^, ^21^ potentially excluding access for individuals with resectable mesothelioma as subsequent drug licensing indications follow the trial entry definitions.

Bevacizumab,^20^ cisplatin with pemetrexed, and nivolumab plus ipilimumab^21^ reduce the risk of death by 23–26% but are only licensed for unresectable disease. The 28% increase in the risk of death with (extended) pleurectomy decortication widens the potential disparity in survival. The increased risk of death reported by trials of maximally cytoreductive approaches such as extended pleurectomy decortication and extrapleural pneumonectomy,^9^ and no survival benefit with lesser approaches in trials of partial pleurectomy, lead us to challenge the concept of resectable mesothelioma.

MARS 2 provided a unique opportunity to evaluate pleurectomy decortication in the context of what were generally considered to be clearly suitable cases for surgery, such as T1–2 N0; uncertain cases, such as N1–2 disease^3^ and biphasic mesothelioma;^22^ and unsuitable cases, such as sarcomatoid disease.^3^ Our inclusion criteria and outcomes of grey and unsuitable were carefully monitored by the data safety and monitoring committee and represented a minority of participants (biphasic 9% and sarcomatoid 3%) in the trial cohort. Although ideally every trial would be perfect by design, in reality, protocol violations can and did occur, with ten (3%) of 335 participants included despite having M1 disease; however, more importantly, violations were balanced, with six in the surgery group and four in the chemotherapy alone group. We believe that these data represent real-life clinical practice. For surgical trials that report harmful or negative outcomes, there is often a tension between trusting randomisation to generate groups that are balanced for measured and unmeasured characteristics, and management up to the point of randomisation such that the results can be taken at face value versus selection and further subselection that could possibly lead to a different conclusion (eg, use of PET-CT, or improvements in surgical staging or techniques with time). We undertook post-hoc analyses, prompted by reviewers' comments, that showed no change in the findings (ie, poorer survival with surgery) despite the use of PET-CT and with time (appendix p 21), which are consistent in the support of randomisation to create balance. We went on further to consider the survival outcome of the ideal selection cohort T1–2 N0 epithelioid-only disease, which still shows poorer survival with surgery (appendix p 22).

Although individuals can anecdotally have non-widespread disease that is conceptually cured by surgical resection, this is the exception rather than the rule and it is not possible to identify such individuals with certainty. The UK mesothelioma investigators have now evaluated all three principal surgical procedures (as detailed by the International Association for the Study of Lung Cancer expert consensus^8^) in randomised trial design settings, reporting insufficient efficacy^23^ and increased risk of death.^9^ Given that both trials of cytoreductive surgery (MARS^9^ and MARS 2) were associated with an increase in death, we question whether radical cytoreductive surgery can continue to be offered outside the governance of a clinical trial.

Potential limitations of MARS 2 include the fewer cycles of chemotherapy and less additional immunotherapy received by participants in the surgery group. The trial design was pragmatic, and this difference is likely to reflect the poor health status after surgery that precluded additional therapies; it is important to recognise that this is part of the intention-to-treat pathway and should be regarded more as an outcome than indicative of group imbalance. Nevertheless, the primary analysis of overall survival indicating poorer survival in the surgery group is robust after adjustment for the differences in chemotherapy and immunotherapies received. Although the trial was not blinded, overall survival is an objective outcome that cannot easily be influenced by bias in reporting. A further limitation is that the ethnicity and gender of participants were not collected.

As with all trials, there could be small differences in baseline characteristics despite randomisation, which will have occurred by chance. To screen for important differences, we calculated standardised mean differences of baseline covariates. All the values were below the 0·2 threshold, indicating only minor differences that are unlikely to have any implication for the results of the trial.

Within the limits of the accuracy of CT staging and assessment, we would expect differences between the clinical T2 subcategories, especially when numbers (denominators) are small and when relative differences can be exaggerated. Given that the principal (parent) T2 category was balanced (21% versus 22%), and as all subcategories carry equal prognostic weight (hence the group T2 assignment), it would not have led to any appreciable imbalance in the prognosis between the two groups, nor any undue effect of risk of death from surgery (especially as the in-hospital mortality for surgery was 4%). The discrepancy between clinical and pathological staging is well known due to the limitation in assessment of disease through imaging modalities alone compared with definitive operative staging, as such upstaging of T and N categories was expected and observed; more importantly, the staging characteristics were balanced at baseline and any limitations of CT staging are the same in both groups.

We did not mandate PET or CT assessment for trial entry in the initial trial design as contrast-enhanced CT was the principal modality used to evaluate resectability. The ASCO^3^ and ESMO^6^ guidelines were published after the start of MARS 2 (June, 2015) and, as the use of PET-CT became more widespread during the course of the trial, by the end, 110 (40%) of 272 participants had PET-CT as part of the work-up (51 [38%] of 135 in the surgery group and 59 [43%] of 137 in the chemotherapy alone group) and our conclusions remained robust to the use of PET-CT (appendix p 21). We also did not mandate invasive mediastinal staging, which was not in routine use at the start of the trial, and subsequent ASCO 2018 recommendations did not contraindicate surgery in the presence of nodal disease (as long as it is given as part of multimodality treatment, which was complied with in the MARS 2 trial).

In conclusion, in this multicentre randomised trial, (extended) pleurectomy decortication and chemotherapy was associated with worse survival, a higher rate of serious adverse events, poorer quality of life, and higher costs among individuals with resectable pleural mesothelioma compared with chemotherapy alone.

## Data sharing

Following publication, anonymised individual participant data will be made available upon request to the corresponding author for secondary research, conditional on assurance from the secondary researcher that the proposed use of the data is compliant with the Medical Research Council Policy on Data Sharing regarding scientific quality, ethical requirements, and value for money. Only data from participants who have consented for their data to be shared with other researchers will be provided.

## Declaration of interests

EL reports grants from Boehringer Ingelheim, Medela, Johnson & Johnson/Ethicon, Covidien/Medtronic, Guardant Health, Takeda, Lilly Oncology, and Bayer, paid to his institution, his company, or personally; consulting fees from BeiGene, Roche, and BMS; honoraria from Medela; two patents (P52435GB and P57988GB) issued to Imperial Innovations; and being founder of My Cancer Companion, Healthcare Companion. SP reports consulting fees from AnHeart Therapeutics, Amgen, AstraZeneca, Bayer, Blueprint, BMS, Boehringer Ingelheim, Ellipses, EQRx, Daiichi Sankyo, GSK, Guardant Health, IO Biotech, Janssen, Lilly, Merck Serono, Mirati, MSD, Novocure, Novartis, PharmaMar, Roche, Takeda, Pfizer, Pierre Fabre, and Turning Point Therapeutics; honoraria from AstraZeneca, Bayer, Guardant Health, Janssen, Merck Serono, Roche, and Takeda; fees for expert testimony from Roche and Merck Serono; support for meeting attendance from Janssen, Roche, and Gilead; and being a member of the British Thoracic Oncology Group, ALK Positive UK, Lung Cancer Europe, Ruth Strauss Foundation, Mesothelioma Applied Research Foundation, and ETOP-IBCSG Partners Foundation Board. NT reports honoraria from BMS. DF reports grants from Astex Therapeutics, Boehringer Ingelheim, Bayer Oncology, Bergen Bio, GSK, MSD, Owkin, Roche, and RS Oncology paid to his institution; consulting fees from MSD, Cambridge Clinical Laboratories, and RS Oncology; honoraria from BMS, BI, MSD, Ikena, and Owkin; and meeting support from RS Oncology and MSD, all paid to Thoracic Oncology Services where he is director. RCR reports research funding from Cancer Research UK, Asthma and Lung UK, June Hancock Mesothelioma Research Fund, Mick Knighton Mesothelioma Research Fund, and Mesobank; and participation on an advisory board for the UK Lung Cancer Coalition. PT reports honoraria from AstraZeneca. LC-S reports support for meeting attendance from Lilly. RC reports honoraria from AstraZeneca, MSD, Takeda, Janssen, Roche, and GSK; support for meeting attendance from Takeda and Janssen; participation on advisory boards for GSK, Takeda, Janssen, Pharmamar, and Amgen; and stock options with TCC and Supportive Care UK. YS reports honoraria from Amgen, AstraZeneca, AbbVie, BMS, MSD, Lilly, Roche, and Takeda; and support for meeting attendance from Takeda and Roche. ZT reports honoraria from AstraZeneca. RS reports honoraria and support for meeting attendance from Lilly and BMS. EF reports membership of the National Lung Cancer and Mesothelioma Clinical Experts Group and Northern Cancer Alliance Targeted Lung Health Check Clinical Lead. All other authors declare no competing interests.
