# Supplementary material for: Extended pleurectomy decortication and chemotherapy versus chemotherapy alone for pleural mesothelioma (MARS 2): a phase 3 randomised controlled trial
Source: Lancet Respir Med. 2024 Jun;12(6):457–66. doi: 10.1016/S2213-2600(24)00119-X (PMC11136673; doi:10.1016/S2213-2600(24)00119-X)
Supplement: Supplementary appendix [file mmc1.pdf]

THE LANCET
Respiratory Medicine

Supplementary appendix

This appendix formed part of the original submission and has been peer reviewed. We post it as supplied by the authors.

Supplement to: Lim E, Waller D, Lau K, et al. Extended pleurectomy decortication and chemotherapy versus chemotherapy alone for pleural mesothelioma (MARS 2): a phase 3 randomised controlled trial. *Lancet Respir Med* 2024; published online May 10. https://doi.org/10.1016/S2213-2600(24)00119-X.

Supplementary Appendix

Contents

List of Investigators and Collaborators ... 2
Trial Methods – additional information ... 9
Statistical Methods – additional information ... 10
Trial Results - supplementary figures ... 13
Trial Results - supplementary tables ... 22
Economic Evaluation Results – additional information ... 32

List of Investigators and Collaborators

Project Management Team Members

Professor Eric Lim, Chief Investigator, Consultant Thoracic Surgeon

Bristol Trials Centre (Main Study)

Professor Chris A Rogers, Methodological lead and Statistician
Kate Ashton, Trial Manager
Dr Barbara Warnes, Trial Manager
Emma Bridgeman, Assistant Trial Coordinator
Katherine Joyce, Assistant Trial Coordinator
Rosie A Harris, Medical Statistician
Dr Nicola Mills, Senior Research Fellow, Quintet Recruitment Intervention
Dr Daisy Elliott, Research Fellow, QuinteT Recruitment Intervention
Dr Nicola Farrar, Research Associate, QuinteT Recruitment Intervention
Dr Elizabeth Stokes, Health Economist
Professor Andrew G Nicholson, Pathologist
Rachel Brophy, Assistant Trial Coordinator
Samantha de Jesus, Assistant Trial Coordinator
Anthanasia Gravani, Assistant Trial Coordinator
Holly McKeon, Assistant Trial Coordinator
Surinder Kaur, Assistant Trial Coordinator
Wendy Underwood, Trial Coordinator

Papworth Trials Unit Collaboration (Pilot Study)

Dr Vikki Hughes, Senior R&D Manager
Jane Elliott, Clinical Project Manager
Claire Matthews, Trial Manager
Phil Noyes, Trial Administrator
Belinda Lees, Trial Manager
Professor Julia Fox-Rushby, Professor of Health Economics

Participating Site Members (ordered by rate of randomisation), n=total recruited

Barts Health NHS Trust (Main study and surgical site, opened June 2017), n=43

Professor Kelvin Lau, Principal Investigator, Consultant Thoracic Surgeon
Mr David Waller, Consultant Thoracic Surgeon
Dr Jeremy Steele, Consultant Medical Oncologist
Joanne Hargrave, Mesothelioma Clinical Nurse Specialist
Professor Peter Szlosarek, Professor of Medical Oncology
Marina Baccarini, Research Nurse
Maria Lapuente, Research Nurse
Eman Backler, Clinical Trials Practitioner
Jenny Moore, Clinical Trials Practitioner
Monalisa Thamed, Clinical Trials Assistant
Pratistha Panday, Clinical Trials Assistant
Resmi Jayachandran, Senior Clinical Trials Practitioner
Maryam Butt, Clinical Trials Assistant
Catherine Norman, Clinical Trials Practitioner
Dr Anju Sahdev, Consultant Radiologist
Dr Ernese Gjafa, Oncologist

Dr Akhila Wimalasingham, Clinical Research Fellow
Veronica Birzu, Clinical Trials Practitioner
Dr Shanthini Crusz, Consultant Medical Oncologist
Faith Dzumbunu, Research Nurse
Shahanara Ferdous, Clinical Trials Practitioner

The Clatterbridge Cancer Centre NHS Foundation Trust (Pilot study site, opened November 2015), n=28

Dr Tony Pope, Principal Investigator, Consultant in Medical Oncology
Dr Carles Escriu, Consultant in Medical Oncology
Dr Richard Griffiths, Consultant in Medical Oncology
Professor Pieter Postmus, Professor of Pulmonary Diseases
Sarah Rose, Research Nurse
Alison Hassall, Advance Research Practitioner
Louisa Brady, Research Nurse
Alana Ross, Clinical Trials Assistant
Masuma Begum, Clinical Trials Assistant
Dr Chloë May, Consultant in Medical Oncology
Dr Ana Ortega, Medical Oncologist
Dr Pavlos Piskilidis, Consultant in Medical Oncology
Dr Jonathan Heseltine, Consultant in Medical Oncology
Maggie Cantrell, Research Nurse
Jennifer Derham, Research Nurse
Emma Barry, Clinical Trials Assistant
Nicholas Garbutt, ECMC Senior Clinical Trials Assistant
Sue Green, Data Manager
Yvonne McCarthy, Senior Clinical Trials Assistant
Jenny Miller, Research Practitioner
Alana Ross, Clinical Trials Assistant

The Beatson West of Scotland Cancer Centre, Greater Glasgow Health Board (Main study site, opened July 2017), n=23

Dr Clinton Ali, Principal Investigator, Consultant Medical Oncologist
Professor Kevin Blyth, Professor of Respiratory Medicine
Kate Beattie, Research Nurse
Gail Lynch, Research Nurse
Austin McInnes, Clinical Trial Coordinator

University Hospitals of Derby and Burton NHS Foundation Trust (Pilot study site, opened November 2015), n=24

Dr Manjusha Keni, Principal Investigator, Consultant Clinical Oncologist
Elizabeth Nadin, Research Practitioner
Dr Andrea Nagy, Consultant Medical Oncologist
Charlotte Downes, Research Nurse
Julie Edmonds, Research Nurse
Christopher Worth, Cancer Clinical Research Nurse Lead
Ellie Piggott, Research Practitioner

The Royal Marsden NHS Foundation Trust (Pilot study site, opened April 2016), n=21

Professor Sanjay Popat, Principal Investigator, Consultant Medical Oncologist
Professor Mary O'Brien, Consultant Medical Oncologist
Dr Nadza Tokaca, Consultant Medical Oncologist

Dr Michael Davidson, Consultant Medical Oncologist
Dr Jaishree Bhosle, Consultant Medical Oncologist
Dr Anna Minchom, Consultant Medical Oncologist
Dr Charlotte Milner-Watts, Clinical Research Fellow
Dr Hazel O'Sullivan, Clinical Research Fellow
Libby Hennessy, Clinical Trials Coordinator
Emma Turay, Clinical Trials Administrator
Bianca Rock, Senior Trials Manager
Agnieszka Yongue, Senior Trials Coordinator
Maria Piga, Research Nurse
Kathy Priest, Research Nurse
Mary Jane de los Reyes Lauigan, Research Nurse
Mariam Mahamood, Clinical Trials Administrator
Dr Nadia Yousaf, Consultant Medical Oncologist

North Bristol NHS Trust (Main study site, opened February 2018), n=15

Professor Nick Maskell, Principal Investigator, Consultant in Respiratory Medicine
Louise Stadon, Research Nurse
Anna Morley, Research Nurse
Dr Duneesha De Fonseka, Respiratory Physician
Dr Amelia Clive, Consultant in Thoracic Medicine
Dr David Arnold, Clinical Lecturer in Respiratory Medicine
Dr Rahul Bhatnagar, Consultant Respiratory Physician
Dr Anna Bibby, Consultant Respiratory Physician
Sarah Smith, Mesothelioma Nurse Specialist
Dr Steven Walker, Registrar in Respiratory Medicine
Natalie Zahan-Evans, Research Nurse
Alice Milne, Respiratory and Renal Administrator

University Hospitals of Leicester NHS Trust (Pilot and surgical site, opened April 2015), n=23

Professor Dean Fennell, Principal Investigator, Chair of Thoracic Medical Oncology
Mr Apostolos Nakas, Consultant Thoracic Surgeon
Mr David Waller, Consultant Thoracic Surgeon
Liz Darlison, Consultant Nurse and Head of Services for Mesothelioma UK
Louise Nelson, Research Nurse
Mr Alan Dawson, Cardiothoracic Surgical Registrar
Dr Margaret Kutka, Consultant Medical Oncology
Rebecca Boyles, Research Nurse
Lydianne Lock, Research Nurse
Maxine Cudbill, Clinical Trials Assistant

Sheffield Teaching Hospitals NHS Foundation Trust (Pilot study and surgical site, opened May 2015), n=19

Mr John Edwards, Principal Investigator, Consultant Thoracic Surgeon
Dr Laura Socci, Consultant Thoracic Surgeon
Ms Sara Tenconi, Consultant Thoracic Surgeon
Helena Stanley, Mesothelioma Clinical Nurse Specialist
Dr Patricia Fisher, Consultant Clinical Oncologist
Professor Matthew Hatton, Consultant Clinical Oncologist
Dr Tathagatha Das, Consultant Clinical Oncologist
Dr Caroline Lee, Consultant Clinical Oncologist
Dr Robin Young, Consultant Medical Oncologist
Dr Emma Bates, Consultant Clinical Oncologist

Hilary Wood, Data Manager
Richard Lloyd, Data Manager
Amy Cooney, Clinical Trials Assistant
Lubna Sheazadi, Data Manager
Chris Blackwell, Clinical Trials Assistant
Helena Hanratty, Research Nurse

Royal Papworth Hospital NHS Foundation Trust (Pilot study site, opened July 2015), n=22

Professor Robert Rintoul, Principal Investigator (from July 2016), Honorary Consultant Respiratory Physician
Kelly Wood, Research Nurse
Amanda Stone, Senior Research Nurse
Kate Slaven, Mesothelioma UK Clinical Nurse Specialist
Amy Gladwell, Clinical Trials Administrator
Suzanne Miller, Clinical Trials Coordinator
C. Jennifer Castedo, Research Nurse
Theresa Green, Research Nurse
Dr David Gilligan, Consultant Clinical Oncologist
Dr Susan Harden, Consultant Clinical Oncologist
Dr Robert Buttery, Consultant Respiratory Physician
Dr David Meek, Consultant Respiratory Physician
Mr Marco Scarci, Principal Investigator (until July 2016), Consultant Thoracic Surgeon

Colchester Hospital University NHS Foundation Trust (Pilot study site, opened November 2015), n=16

Dr Charlotte Ingle, Principal Investigator (from April 2019), Consultant Clinical Oncologist
Dr Dakshinamoorthy Muthukumar, Principal Investigator (until April 2019), Consultant Oncologist
Celine Driscoll, Senior Therapeutic Radiographer
Katrina Cooke, Research Nurse
Hayley Hewer, Research Nurse
Liz Hunting, Research Nurse
Michelle Marshall, Research Nurse
Louise Mabelin, Research Nurse
Andrea Nears, Research Nurse
Lucy Thorogood, Research Coordinator
Peter Tovey, Data Administrator

Manchester University NHS Foundation Trust (Pilot study site, opened December 2015), n=17

Dr Paul Taylor, Principal investigator, Consultant in Medical Oncology
Dr Laura Cove Smith, Consultant in Medical Oncology
Dr Raffaele Califano, Consultant in Medical Oncology
Dr Yvonne Summers, Consultant in Medical Oncology
Sara Waplington, Clinical Trials Coordinator
Lyndsey Holt, Research Nurse
Maria Blinston, Research Nurse
Juliette Novasio, Clinical Trials Manager
Amal Ismail, Clinical Trials Administrator
Susannah Moss, Clinical Trials Administrator
Ikraam Warsame, Clinical Trials Administrator

Norfolk and Norwich University Hospitals NHS Foundation Trust (Main study site, opened June 2018), n=10

Dr Zacharias Tasigiannopoulos, Principal Investigator, Consultant Clinical Oncologist
Dr Eleanor Mishra, Consultant Respiratory Physician

Dr Pinelopi Gkogkou, Consultant Clinical Oncologist
Adela Dann, Clinical Trials Practitioner
Susan Halliwell-Bass, Research Administrator
Mark Harmer, Research and Trials Practitioner

Guy's and St. Thomas' NHS Foundation Trust (Main study and surgical site, opened August 2018), n=10

Mr Andrea Bille, Principal Investigator, Consultant Thoracic Surgeon
Professor James Spicer, Professor of Experimental Cancer Medicine and Consultant in Medical Oncology
Dr Eleni Karapanagiotou, Consultant in Medical Oncology
Catherine Andaya, Research Nurse
Annie Rose Henry, Research Nurse
Saoirse Daly, Research Nurse
Emma Helliwell, Research Nurse
Francesca Curran, Research Nurse
Amy Quinn, Clinical Research Nurse
Jehan Mansi, Clinical Trials Practitioner

Maidstone and Tunbridge Wells NHS Trust (Main study site, opened October 2018), n=9

Dr Riyaz Shah, Principal Investigator, Consultant Medical Oncologist
Eirini Petroyannou, Research Nurse
Deborah Wilcox, Research Nurse
Dr Parvin Begum, Medical Oncologist
Dr Maria Karina, Consultant Medical Oncologist
Dr Samantha Kestenbaum, Medical Oncologist
Dr Simon Rodney, Medical Oncologist
Dr Alicja Synowiec, Oncologist – Clinical Trials
Dr Zayd Tripp, Medical Oncologist
Dan Gorman, Research Nurse
Eve McGarry, Research Nurse
Sophy Mount, Research Nurse
Monica Tavares Barbosa, Research Nurse
Alison Davison, Clinical Trials Coordinator for Lung
Laura Edwards, Clinical Trials Administrator
Iveta Los, Clinical Trials Administrator
Suzanne Mellish, Clinical Trials Administrator
Angela Percival, Clinical Trials Coordinator for Lung
Ruth Perry, Clinical Trials Administrator
Kimberley Snoad, Clinical Trials Administrator
Deborah Webber, Clinical Trials Coordinator for Lung

South Tyneside and Sunderland NHS Foundation Trust (Pilot study site, opened June 2015), n=14

Dr Andrew McNair, Principal Investigator (from March 2022), Consultant Respiratory Physician
Dr Liz Fuller, Principal Investigator (until March 2022), Consultant Respiratory Physician
Dr Chris Jones, Consultant Medical Oncologist
Dr Adam Hassani, Consultant Clinical Oncologist
Dr Rhona McMenemin, Consultant Clinical Oncologist
Dr Thomas Ross, Respiratory Research Fellow
Charlotte Caroline, Research Nurse
Jane Cole, Research Nurse
Nadia Elkaram, Research Nurse
Amy Burns, Clinical Trials Coordinator
Judith McKenna, Clinical Trials Officer

Tracey Lowdon, Data Manager

Barking, Havering and Redbridge University Hospitals NHS Trust (Main study site, opened July 2018), n=6

Dr Jonathan Shamash, Principal Investigator, Consultant Medical Oncologist
Alison Ray, Clinical Trials Nurse
Charlotte Westley, Research Nurse
Helen Mackenzie, Clinical Research Assistant

South Tees Hospitals NHS Foundation Trust (Pilot study site, opened November 2015), n=8

Dr Talal Mansy, Principal Investigator, Consultant Medical Oncologist
Charlotte Jacobs, Clinical Trials Coordinator
Andrea Watson, Clinical Trials Coordinator
Dr Sarah Essex, Clinical Trial Manager
Dr Louise Li, Consultant Medical Oncologist
Dr Eleanor Aynsley, Consultant Clinical Oncologist
Leeds Teaching Hospitals NHS Trust (Pilot study site, opened December 2015), n=4

Mr Richard Milton, Principal Investigator, Consultant Thoracic Surgeon
Victoria Ashford-Turner, Research Nurse

The Royal Wolverhampton NHS Trust (Pilot study site, opened January 2016), n=7

Dr Pek Koh, Principal Investigator (from October 2017), Consultant Clinical Oncologist
Mr Ian Morgan, Principal Investigator (until October 2017), Consultant Cardiothoracic Surgeon
Victoria Lake, Research Nurse
Elizabeth Radford, Clinical Trials Assistant
Rachel Pearse, Research Nurse
Katherine Vassell, Research Nurse

Royal Gwent Hospital, Aneurin Bevan University Health Board (Pilot study site, opened February 2016), n=7

Dr Andreea Alina Ionescu, Principal Investigator, Consultant Respiratory Physician
Simon Hodge, Research Nurse
Dr Paul Shaw, Consultant Clinical Oncologist
Evelyn Baker, Research Nurse
Sean Cutler, Trial Coordinator
Maxine Nash, Trial Coordinator
Jemma Tuffney, Trial Coordinator

Peterborough City Hospital, North West Anglia NHS Foundation Trust (Pilot study site, opened May 2015), n=4

Dr Sarah Treece, Principal Investigator, Consultant Clinical Oncologist
Dr Abigail Hollingdale, Consultant Clinical Oncologist
Holly Warman, Clinical Trials Assistant
Emma Ingall, Clinical Trials Assistant
Claire Palombo, Clinical Trials Assistant
Chloe Eddings, Research Nurse
Terri-Anne Baker, Research Nurse
Claire Snowden, Research Nurse
Helen Bowyer, Research Nurse
Kerrie Cavanagh, Administrator

University Hospitals Plymouth NHS Trust (Main study site, opened July 2018, n=3

Dr Amy Roy, Principal Investigator, Consultant Clinical Oncologist
Dr Bojidar Goranov, Consultant Clinical Oncologist
Dr Sarah Prance, Clinical Trial Doctor
Rebecca Baker-Petley, Research Nurse
Hilary Congdon, Research Nurse
Helen Davies, Research Nurse
Laura Evenden, Research Nurse
Jenny Fisher, Research Nurse
Irene Harvey, Research Nurse
Amanda Hind, Research Nurse
Matthew Mills, Research Nurse
Julie Pascoe, Research Nurse
Olivia Reed-Poysden, Research Nurse
Bridget Aire, Clinical Trials Assistant
Lucy Cadmore, Clinical Trials Assistant
Kay Facey, Senior Research Administrator
Julia Jaczo, Data Manager
Emily Sutton, Data Manager
Heidi Tunnicliffe, Data Manager

University Hospitals Birmingham NHS Foundation Trust (Main study site, opened October 2019), n=2

Professor Gary Middleton, Principal Investigator, Professor in Medical Oncology
Parminder Sohal, Oncology Research Sister
Richard Winter, Data Manager
Leila Baghdad, Clinical Trials Coordinator
Dr Haider Abbas, Consultant Medical Oncologist
Dr Philip Earwalker, Consultant Medical Oncologist
Donna Gillen, Research Nurse
Sharon Hackett, Research Nurse
Stephanie Palmer, Research Nurse
Catherine Prest, Research Nurse
Nafeesah Ahmad Haider, Lead Data Manager
Kam Gareja, Data Manager
Aliyah Mannan, Project Manager
Dr Deborah Tattersall, Consultant Radiologist

Glasgow Jubilee National Hospital (Surgical site, opened July 2017)

Mr Alan Kirk, Principal Investigator, Consultant Thoracic Surgeon
Mr Rocco Bilancia, Consultant Thoracic Surgeon
Elizabeth Boyd, Research Nurse
Julie Buckley, Research Nurse
Christine Aitken, Research Nurse

Membership of Independent Oversight Committees

Independent Trial Steering Committee Members

Professor Marcus Flather (Chair), Clinical Professor in Medicine
Dr Pauline Leonard (Interim Chair until November 2019), Consultant Medical Oncologist
Professor Tom Treasure (Previous Chair), Professor of Cardiothoracic Surgery
Dr Paul Beckett, Consultant Respiratory Physician

Professor Fergus Gleeson, Professor of Radiology and Consultant Radiologist
Dr Fergus Macbeth, Retired Clinical Oncologist
Hon Dr Mavis Nye, Patient Representative
Professor Harvey Pass, Professor of Thoracic Oncology
Professor Rolf Stahel, Chair Comprehensive Cancer Center
Miss Carol Tan, Consultant Thoracic Surgeon

Independent Data Monitoring and Safety Committee Members

Professor Linda Sharples (Chair), Professor of Medical Statistics
Professor Mark Britton, Consultant Physician
Professor Joseph Friedberg, Professor of Surgery and Surgeon-in-Chief
Professor Robin Rudd, Consultant Physician
Professor Valerie Rusch, Thoracic Surgeon
Professor Peter Goldstraw, Emeritus Professor of Thoracic Surgery (until May 2018)

Trial Methods – additional information

Inclusion criteria

Patient may enter study if ALL of the following apply:

1. 16 years of age or over
2. Tissue (cytology or histology) confirmed epithelioid, sarcomatoid or biphasic mesothelioma*
3. Disease confined to one hemi-thorax based on CT assessment
4. Disease deemed surgically resectable**
5. Fit for surgery**
6. Capacity to provide written informed consent to participate in the trial

*The "diagnosis" of mesothelioma is based on cytology and / or histopathology results as reviewed by MDT to be of sufficient certainty to recommend chemotherapy as treatment.
**To be confirmed by a surgeon at a MARS 2 surgical site

Exclusion criteria

Patient may not enter study if ANY of the following apply:

1. Severe shortness of breath (this is defined as an Eastern Cooperative Oncology Group (ECOG) status ≥ 2, or if lung function tests are performed: pre-operative forced expiratory volume after one second (FEV1) or transfer factor of the lung for carbon monoxide (TLco) less than 20%);
2. Serious concomitant disorder that would compromise participant safety during surgery (e.g. evidence of end organ failure)
3. Severe heart failure (this is defined as NYHA III or IV or if an echocardiogram is performed an ejection fraction less than 30%)
4. End stage kidney failure requiring dialysis
5. Liver failure (e.g. encephalopathy and/or coagulation abnormalities)
6. Prisoner
7. Patient lacks capacity to consent
8. Existing co-enrolment in another interventional clinical trial that aims to improve survival

Histology quality assurance procedure

For pathology quality assurance purposes in MARS 2 the following histology samples, collected as part of standard care for diagnostic purposes, were reviewed by an Independent Pathologist:

- 10% of all histology samples (both pre- and post-operatively)
- All samples deemed 'unable to classify' at baseline and post-operatively.
- All samples where the histology sub-diagnosis varied unexpectedly pre- and post-operatively.

All physical slides or digital images prepared and used to confirm the mesothelioma histology sub-group classification at baseline or post-operatively were submitted for review by the Independent Pathologist.

Statistical Methods – additional information

Sample size calculation

The target 30% reduction in risk of death (hazard ratio 0.7) with surgery equated to a median survival of 24 months in the surgery group compared to the assumed 16.8 months on the chemotherapy alone group. The sample size was calculated using the PASS software version 14.0 (NCSS statistical software, LLC, East Kaysville, Utah) assuming the data would be analysed using a two-sided log-rank test.

All outcomes

Analyses were adjusted for the minimisation factors of age, performance status and cell type as fixed effects, and site was fitted as a random effect where possible.

Analysis of time-to-event outcomes

In the analysis of overall survival (OS), the proportional hazards assumption was violated due to treatment group for univariate and multivariate models, as indicated by the crossing of the survival curves, and confirmed using estat phtest in Stata (P value from multivariate model, p=0.014). Therefore, the difference in restricted mean survival time (RMST) to 24 months was calculated, and for the Cox proportional hazards regression analyses the survival time was split into two periods, from randomisation to the point the Kaplan-Meier survival curves crossed (42 months) and after the point of crossover. A treatment by period interaction was added to the model to enable the epoch-specific treatment effect to be calculated.

Progression-free survival (PFS) was defined as the time from randomisation to disease progression or death from any cause. In the analysis of PFS, the proportional hazards assumption was violated due to cell type. Therefore, a stratified Cox proportional hazards model was used (stratified by cell type).

Cox regression was used for all subgroup, sensitivity, and exploratory analyses as it allowed for both interaction terms used to compare subgroups, and it supported the pre-specified modelling strategies used in the sensitivity and exploratory analyses (see below for details). Software used for analysis of RMST did not support these strategies.

For both OS and PFS, a clustered sandwich estimator was used in the Cox proportional hazards model to adjust the standard errors for clustering within sites, as shared frailty terms did not converge. The calculation of RMST did not adjust for site.

There were no missing data for survival outcomes; all patients were included in the models up until the time of their last follow up at which point, they were censored (if they had not experienced the event of interest).

Analysis of binary outcomes

Binary safety outcomes were analysed using mixed effects generalised linear models with Poisson family, log link and robust standard errors. There were no missing data for binary outcomes; models were adjusted for time followed up in the study by adding the log of exposure time as an offset term.

Analysis of count outcomes

Count outcomes were analysed using mixed effects generalised linear models with Poisson family and log link, or negative binomial family and log link if data were over-dispersed. There were no missing data for count outcomes; models were adjusted for time followed up in the study by adding the log of exposure time as an offset term.

Analysis of HRQoL outcomes

No restriction on the time window for each HRQoL time point was specified in the protocol. The distribution of time windows for each HRQoL time point were examined by group; the distributions were balanced across treatment groups and therefore data from all time points were included in the models.

HRQoL outcomes were measured at consent, randomisation, and subsequent follow-up visits. The randomisation HRQoL score was modelled as a covariate where available, otherwise the HRQoL score at consent was used. EQ-5D scores were calculated using the Stata command eq5dmap[1].

For each QLQ-C30 scale score, missing baseline scores were imputed using the median baseline score for the cohort as a whole, as fewer than 5% of participants with at least one post-randomisation score were missing baseline scores. Multiple imputation was used to impute missing baseline EQ-5D scores using 20 imputed datasets. Missing post-randomisation HRQoL scores were not imputed as data could be assumed to be missing at random. There is negligible benefit in imputing missing outcome data if it can be assumed to be missing at random.[1]

Strategy for modelling longitudinal HRQoL outcomes in order of preference: 1) joint longitudinal survival model; 2) linear mixed effects model (used if the joint longitudinal survival model did not provide an adequate fit or there were convergence issues); 3) mixed effects ordinal logistic regression model (used if the previous models were not appropriate, the HRQoL score only took four possible values and the proportional odds assumption held); 4) mixed effects logistic regression models (used if the proportional odds assumption did not hold for time or treatment variables or the ordinal logistic regression model did not converge).

A time by treatment interaction was added to all models; overall treatment effects are presented unless the interaction reached 10% statistical significance, in which case treatment effects for each time point are provided.

Outcomes	Model used	Time adjustment	Survival adjustment	Effect reported
QLQ-C30 global health status, physical functioning, role functioning, cognitive functioning, and emotional functioning scores	Joint longitudinal survival model	Fixed or random depending on model fit assessed using likelihood ratio tests	Survival time modelled jointly with HRQoL score	Mean difference
EQ-5D, QLQ-C30 social functioning, fatigue, and pain scores	Linear mixed effects model	Fixed; different variance/covariance structures assessed using likelihood ratio tests	EQ-5D score of 0 imputed after death; no adjustment for other outcomes	Mean difference
QLQ-C30 dyspnoea, insomnia, and constipation scores	Ordinal logistic regression	Fixed	None	Odds ratio
QLQ-C30 diarrhoea, nausea, appetite loss, and financial difficulties scores	Logistic regression	Fixed	None	Odds ratio

Exploratory analyses examining the effect of surgical site expertise and surgeon

In the exploratory analyses investigating the effect of surgical site expertise and surgeon, surgical site and surgeon were defined based on the surgical site/surgeon who performed the operation. Participants who did not receive surgery were grouped together in one 'chemotherapy only' group.

Sensitivity analyses adjusting primary outcome for number of first-line chemotherapy cycles received

Two sensitivity analyses investigating the impact of number of first-line chemotherapy cycles were performed by adding an indicator to the primary outcome model as a time-varying covariate (TVC). The indicator switched on (=1) once the participant started receiving first-line chemotherapy post-randomisation and switched off (=0) after first-line chemotherapy ended plus a specified interval.

Two definitions of the interval were used.

In the first analysis, indicated by * in Figure 4, the interval was defined as 4x the average half-life of the chemotherapy drugs received.

- Cisplatin half-life = 36 to 47 days[2]; interval = 41.5 days x 4 = 166 days
- Carboplatin half-life = 2.6 to 5.9 hours[3]; interval = 4.25 hours x 4 = 0.71 days
- Pemetrexed half-life = 3.5 hours[4]; interval = 3.5 hours x 4 = 0.58 days

If a participant did not receive Cisplatin, Carboplatin or Pemetrexed, the indicator was switched off one day after chemotherapy ended.

In the second analysis, indicated by ** in Figure 4, the indicator was switched off 21 days after chemotherapy ended.

Sensitivity analyses examining effect of non-adherence with the intervention

The three assumptions of the IV analyses used to estimate the complier average causal effect were: 1) The instrument (randomisation group) was associated with the exposure (treatment received), 2) the instrument was independent of confounding factors between exposure and outcome, and 3) the instrument only affected the outcome through influencing the exposure. These assumptions were likely to be met due to the instrument being randomised group.

Sensitivity analyses adjusting for additional treatments received

A sensitivity analysis investigating the impact of additional treatments received was performed by adding an indicator to the primary outcome model as a TVC. The TVC was switched to (1) if the participant received immunotherapy or another intervention with an established effect on overall survival either in a clinical trial or as part of standard care (e.g. pembrolizumab, nivolumab, bevacizumab), or (2) if the participant received an intervention in another clinical trial where the effect on overall survival is unknown, or where it is known to have no effect on overall survival. The TVC was mutually exclusive; patients were grouped under (1) if they met criteria 1, or under (2) if they met criteria 2 and did not meet criteria 1. Participants who did not meet the criteria for any of the above were grouped together. The indicator switched on once the participant started receiving treatment that met the criteria for the above categories (once a participant met criteria 1, the TVC remained 1 throughout).

Outcome	Number of participants included in analysis, n(%)
Overall survival (primary)	335 (100%)
Progression-free survival	335 (100%)
Total number of CTCAE 3+ events	335 (100%)
Any CTCAE 3+ event within each MedDRA soc	335 (100%)
QLQ-C30 global health status, physical, social, role, and cognitive functioning, diarrhoea, fatigue, nausea, pain, dyspnoea, insomnia, appetite, constipation, financial difficulties	273 (81%)
QLQ-C30 emotional functioning	272 (81%)*
EQ-5D	324 (97%)†

* One patient only had one post-randomisation QoL score which was considered an outlier and was therefore excluded from the analysis.
† EQ-5D was only collected for part of the study

References

1. Twisk J et al. Multiple imputation of missing values was not necessary before performing a longitudinal mixed-model analysis. J Clin Epidemiol 2013; 66(9): 1022-8.

Trial Results - supplementary figures

Figure S1 Any CTCAE grade 3+ event within each MedDRA system organ class

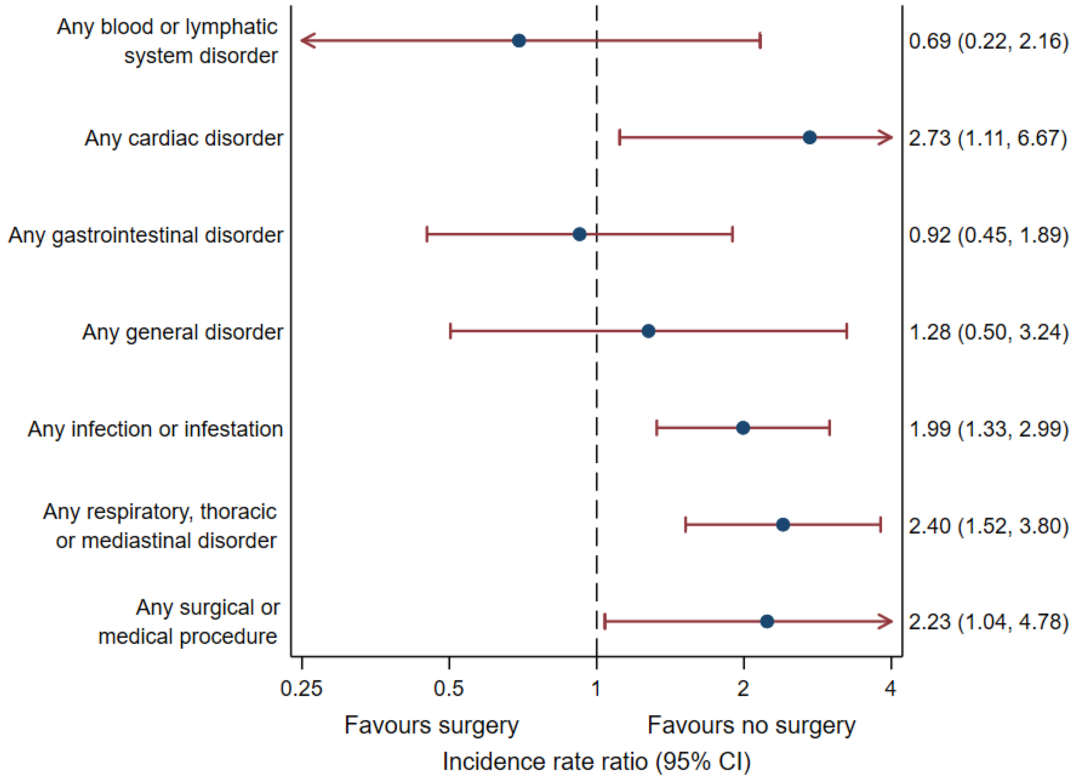

Figure S2 Any CTCAE grade 3+ event within each MedDRA system organ class using randomised group as an instrumental variable

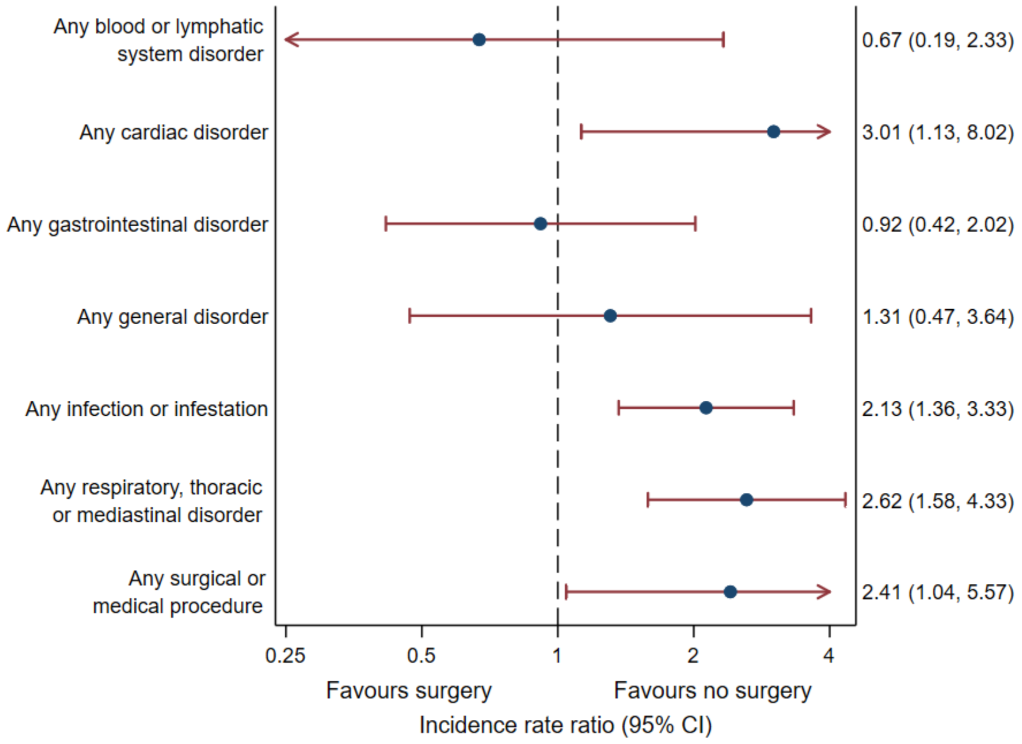

Figure S3 QLQ-C30 physical functioning scores over time

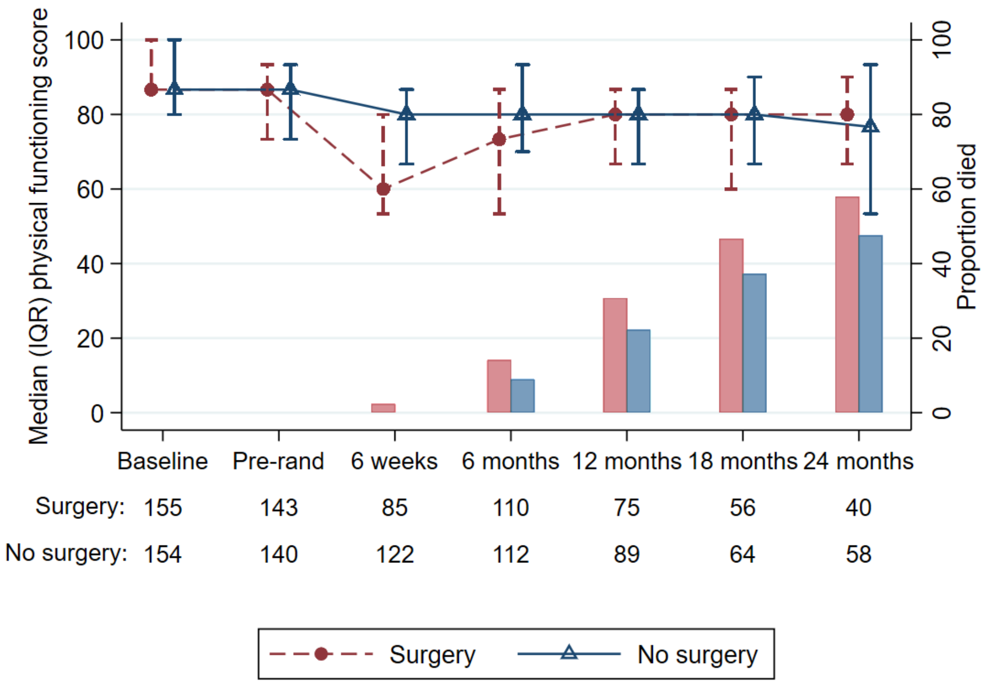

Scores range from 0 to 100. Higher scores indicate better health.

Figure S4 QLQ-C30 social functioning scores over time

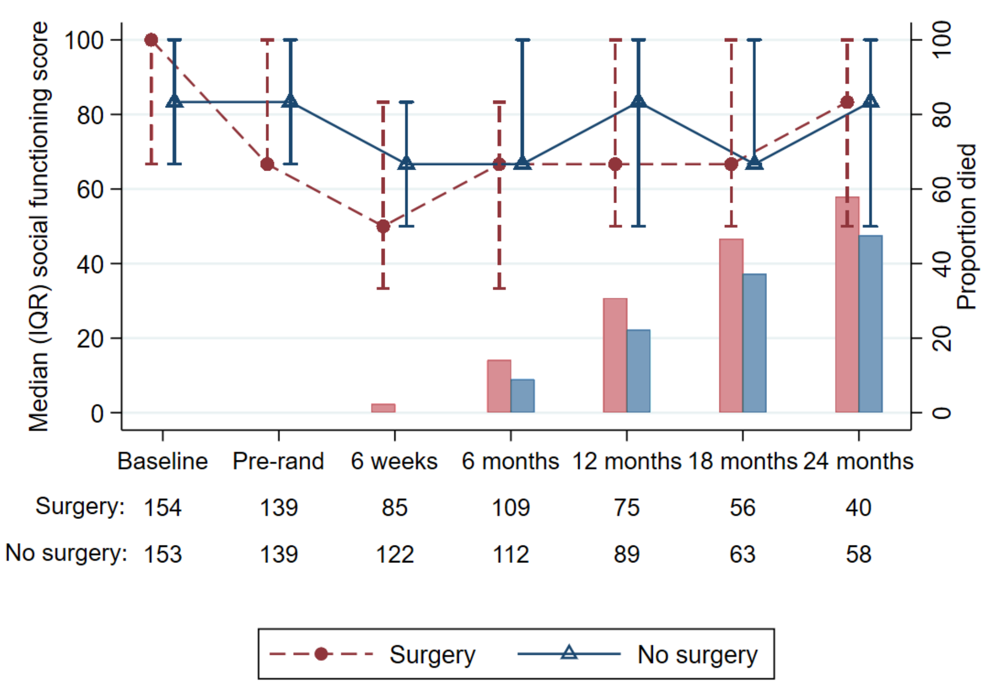

Scores range from 0 to 100. Higher scores indicate better health.

Figure S5 QLQ-C30 role functioning scores over time

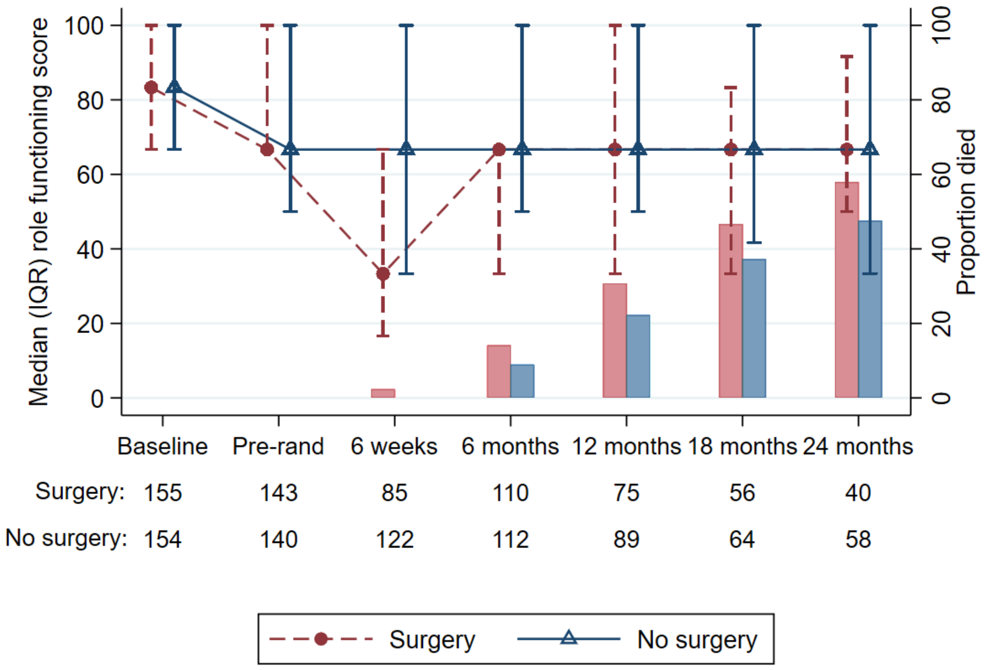

Scores range from 0 to 100. Higher scores indicate better health.

Figure S6 QLQ-C30 cognitive functioning over time

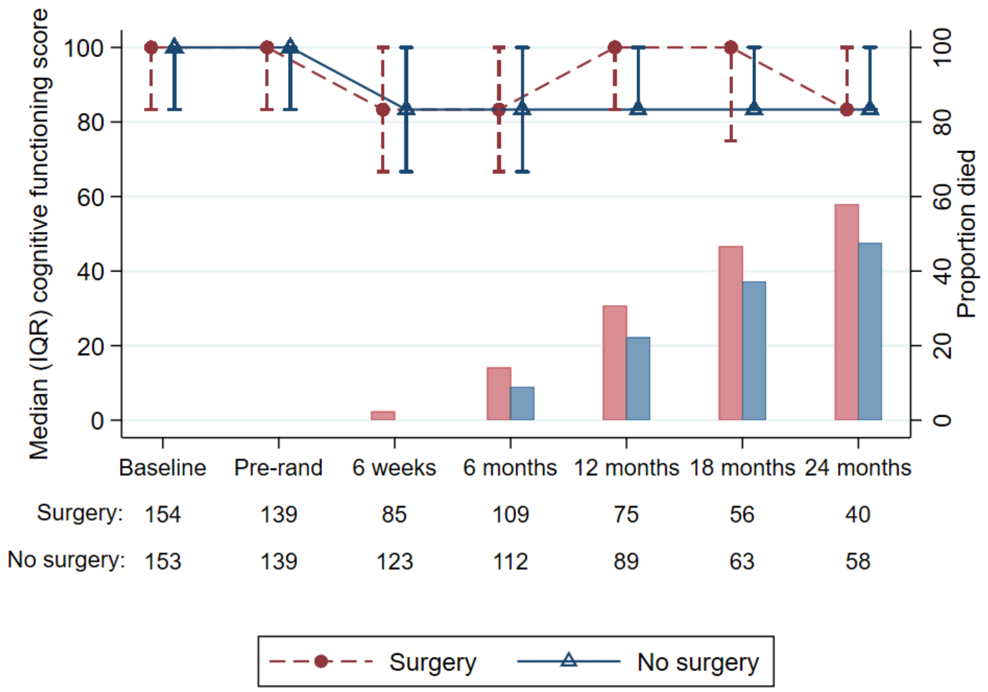

Scores range from 0 to 100. Higher scores indicate better health.

Figure S7 QLQ-C30 emotional functioning scores over time

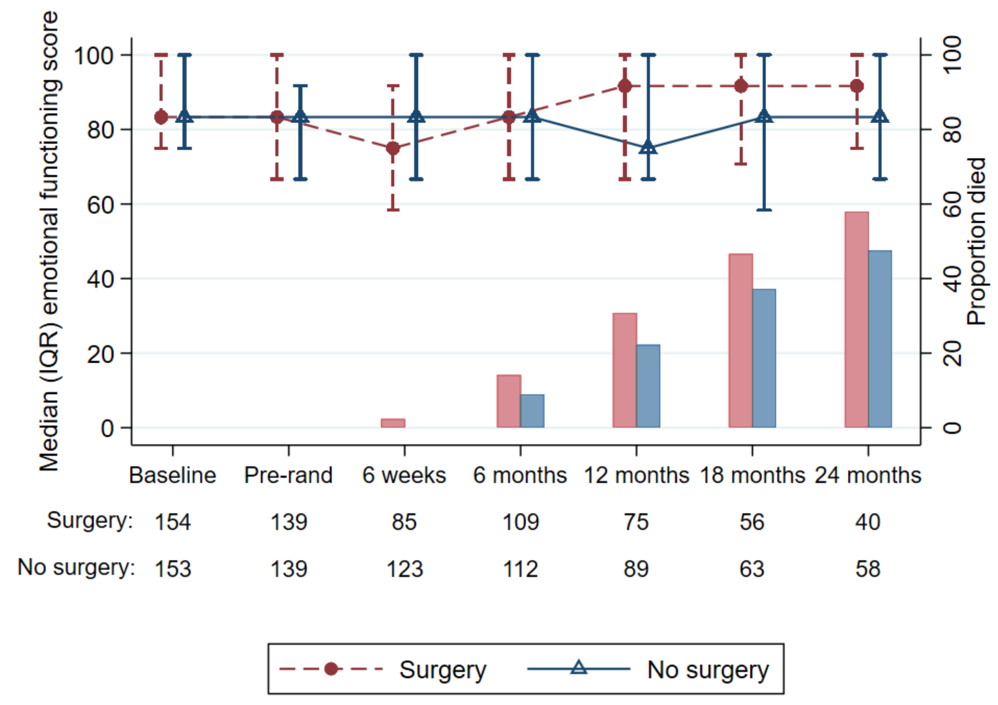

Scores range from 0 to 100. Higher scores indicate better health.

Figure S8 QLQ-C30 diarrhoea symptoms over time

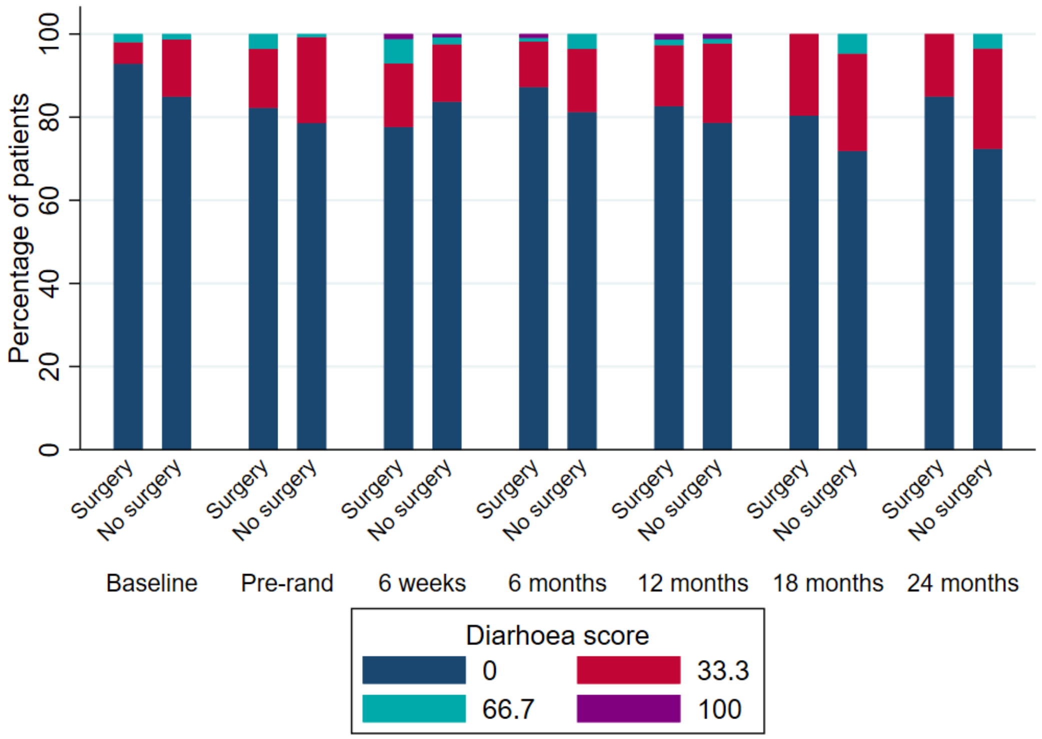

Scores range from 0 to 100. Higher scores indicate more symptoms.

Figure S9 QLQ-C30 fatigue symptom scores over time

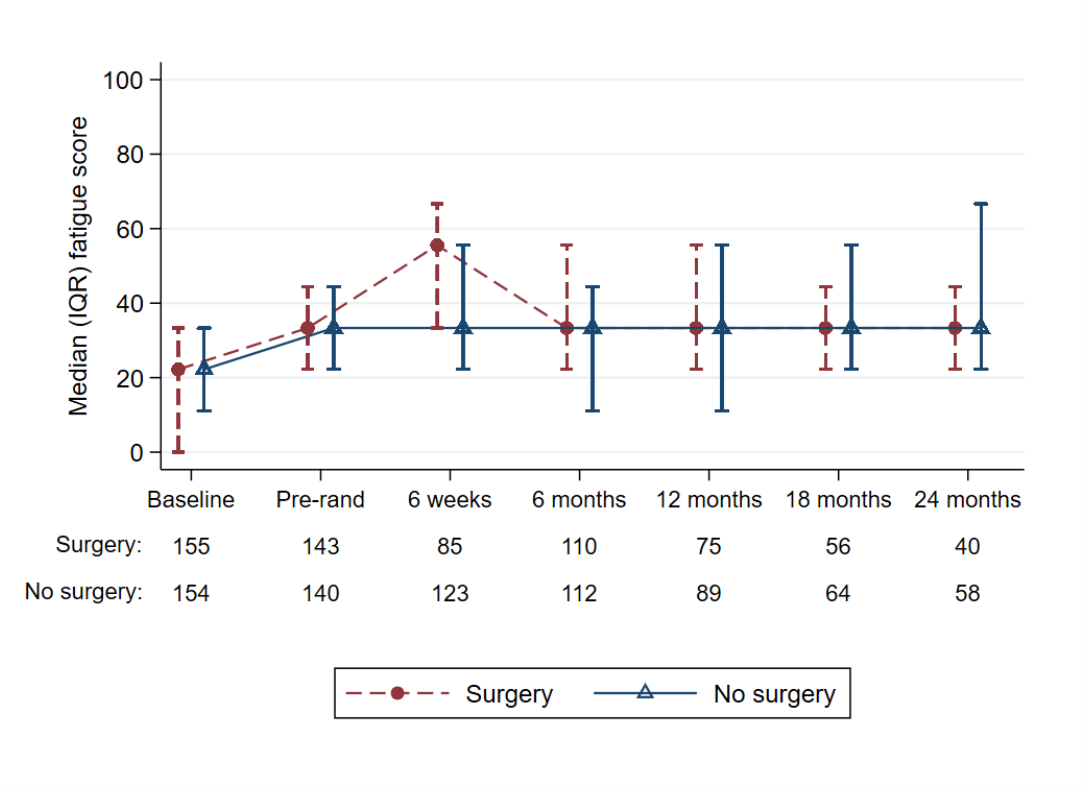

Scores range from 0 to 100. Higher scores indicate more symptoms.

Figure S10 QLQ-C30 nausea symptom scores over time

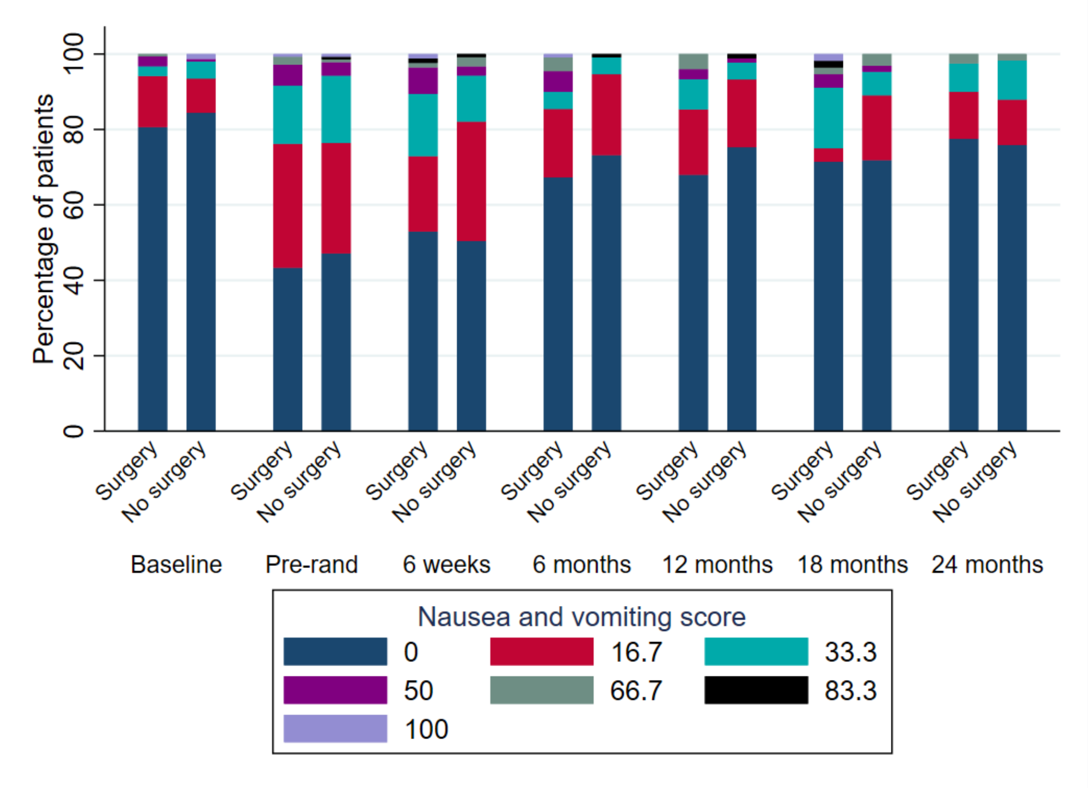

Scores range from 0 to 100. Higher scores indicate more symptoms.

Figure S11 QLQ-C30 pain scores over time

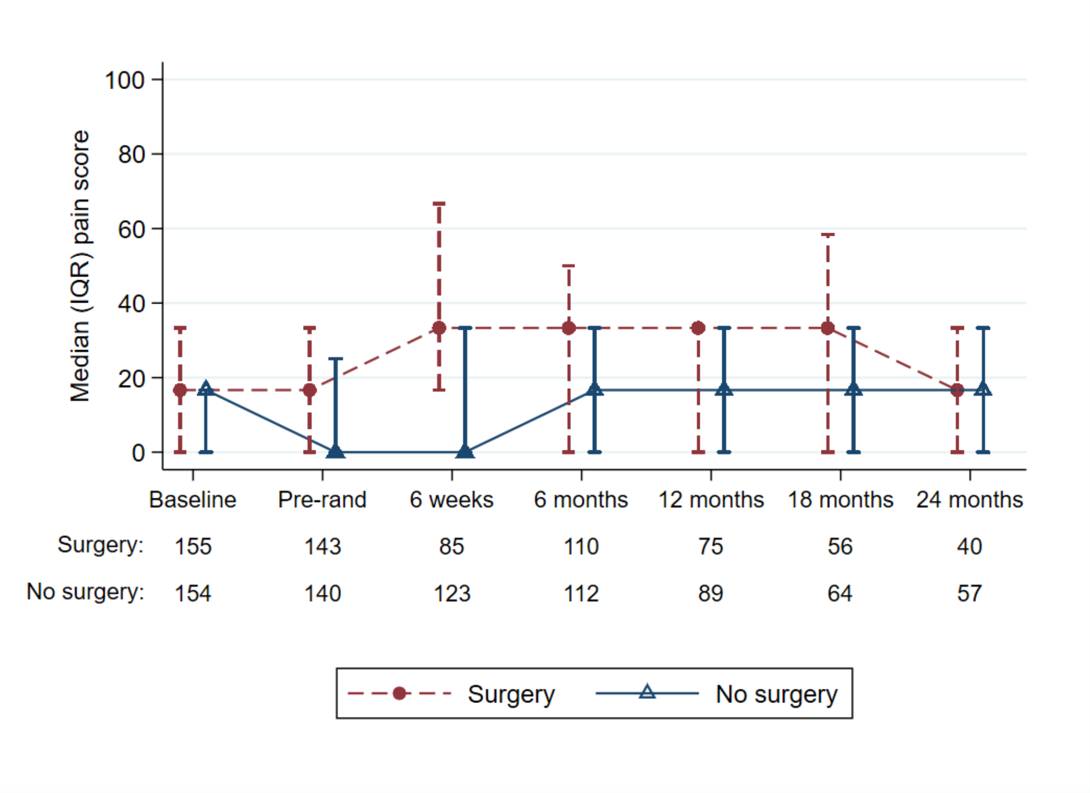

Scores range from 0 to 100. Higher scores indicate more symptoms.

Figure S12 QLQ-C30 dyspnoea symptom scores over time

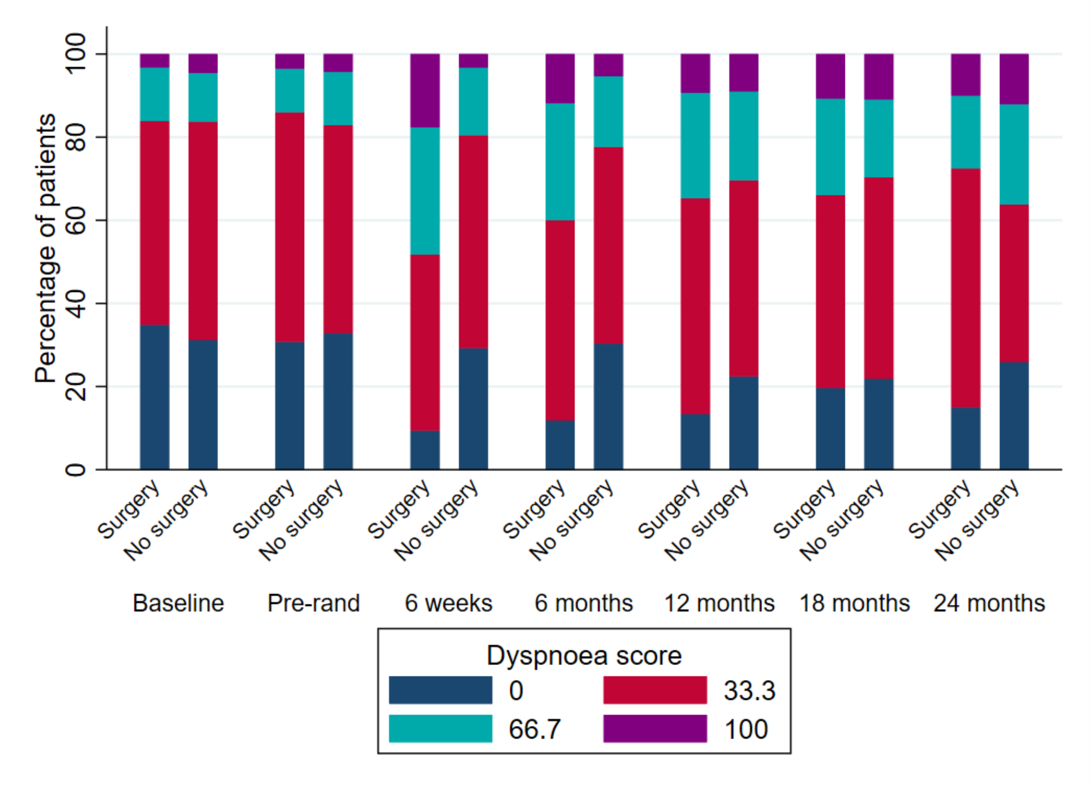

Scores range from 0 to 100. Higher scores indicate more symptoms.

Figure S13 QLQ-C30 insomnia symptom scores over time

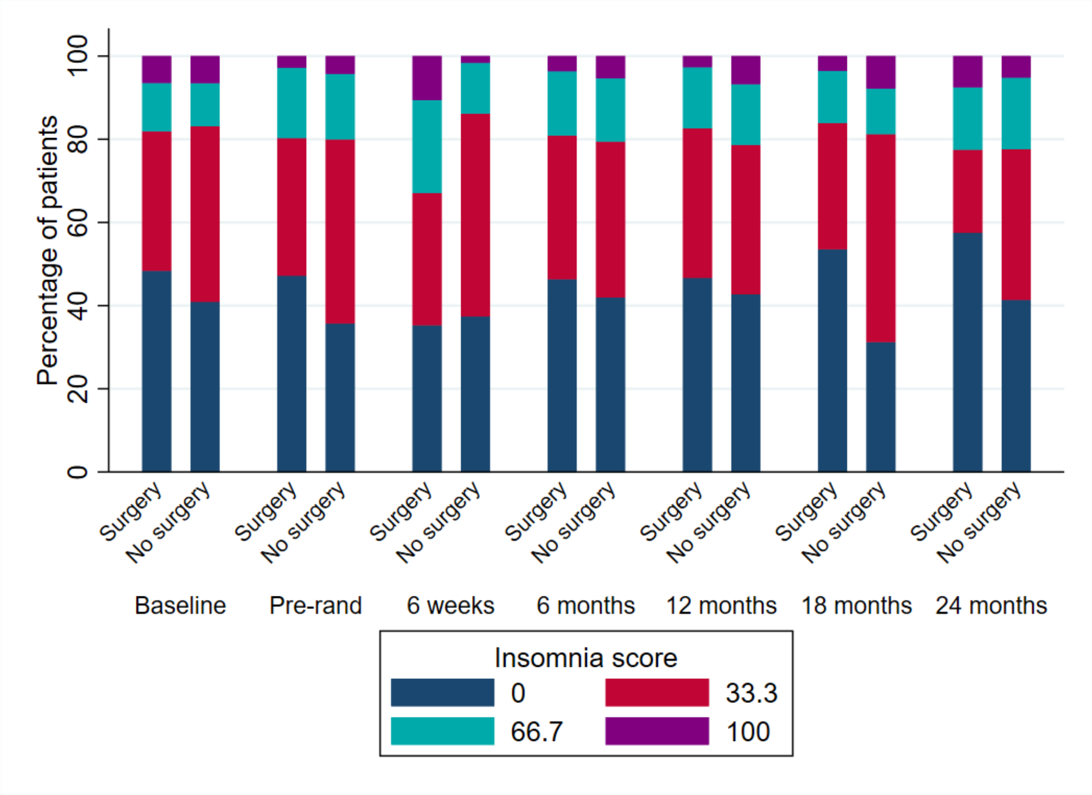

Scores range from 0 to 100. Higher scores indicate more symptoms.

Figure S14 QLQ-C30 loss of appetite symptom scores over time

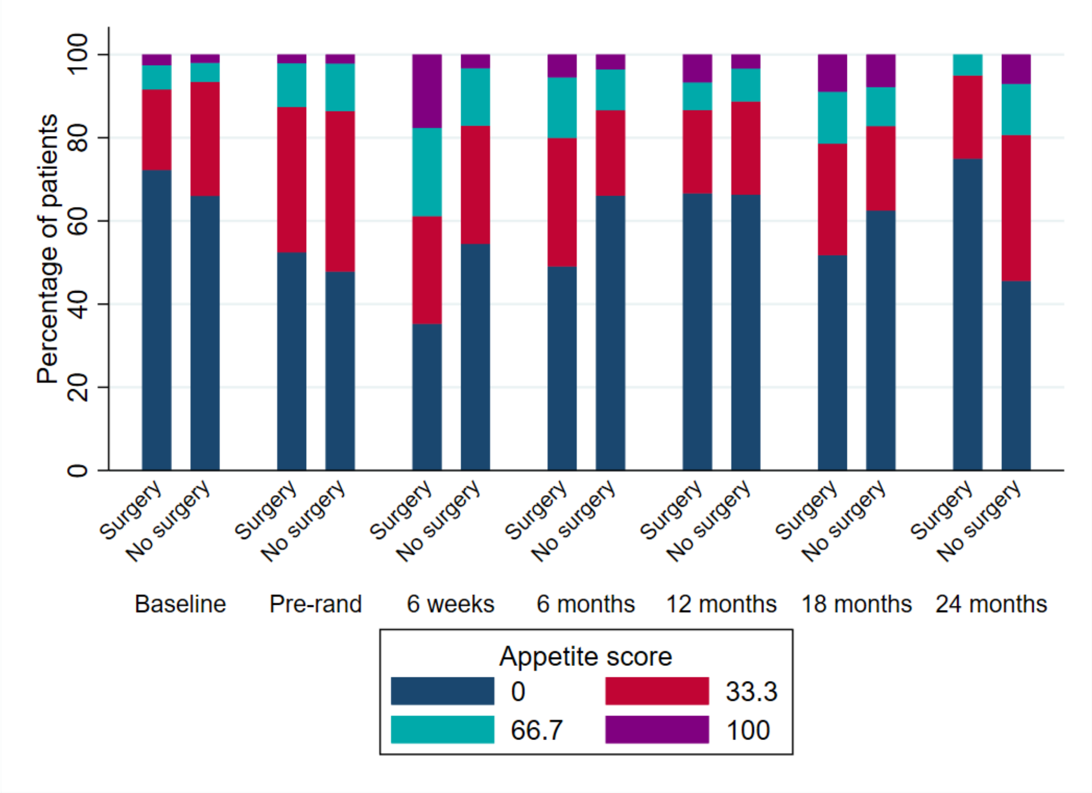

Scores range from 0 to 100. Higher scores indicate more symptoms.

Figure S15 QLQ-C30 constipation symptom scores over time

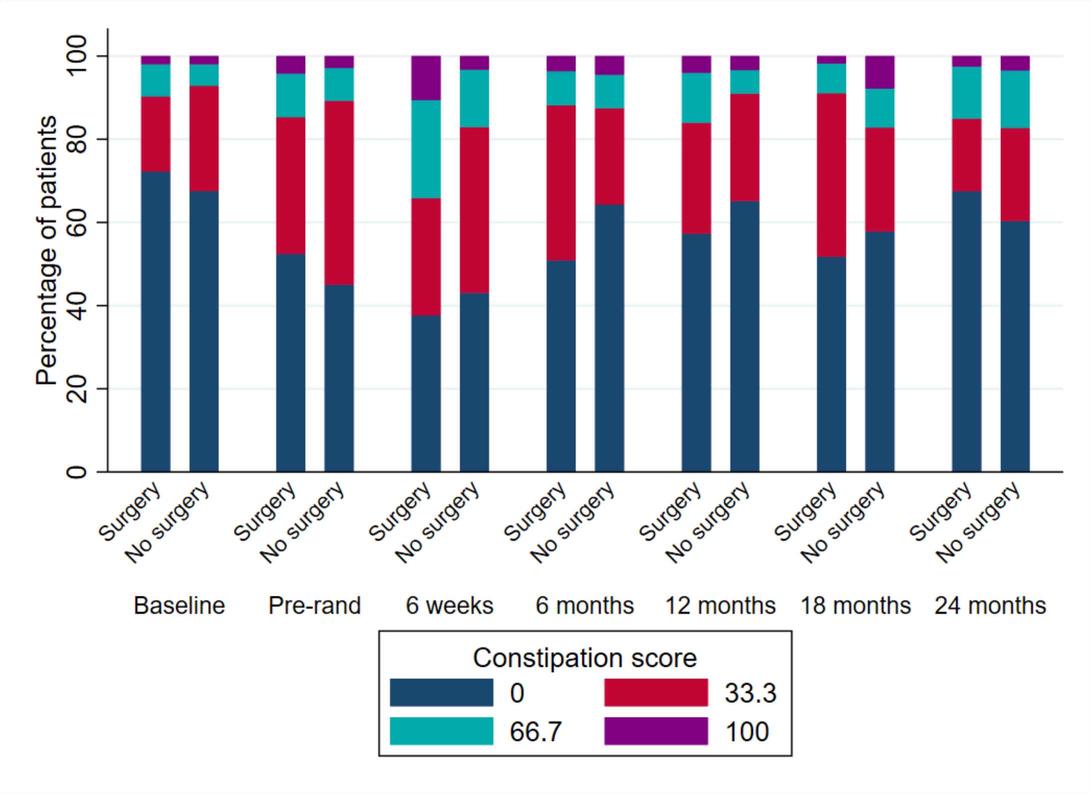

Scores range from 0 to 100. Higher scores indicate more symptoms.

Figure S16 QLQ-C30 financial difficulty scores over time

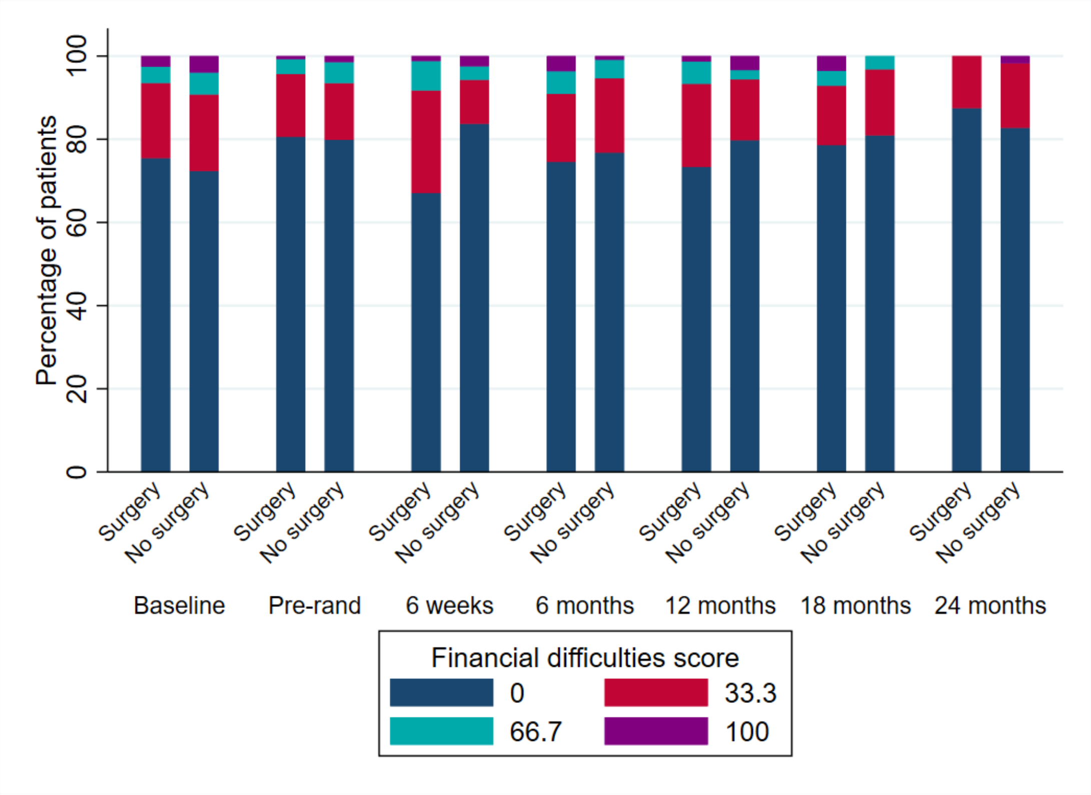

Scores range from 0 to 100. Higher scores indicate more symptoms.

Figure S17 Overall survival by PET-CT subgroups

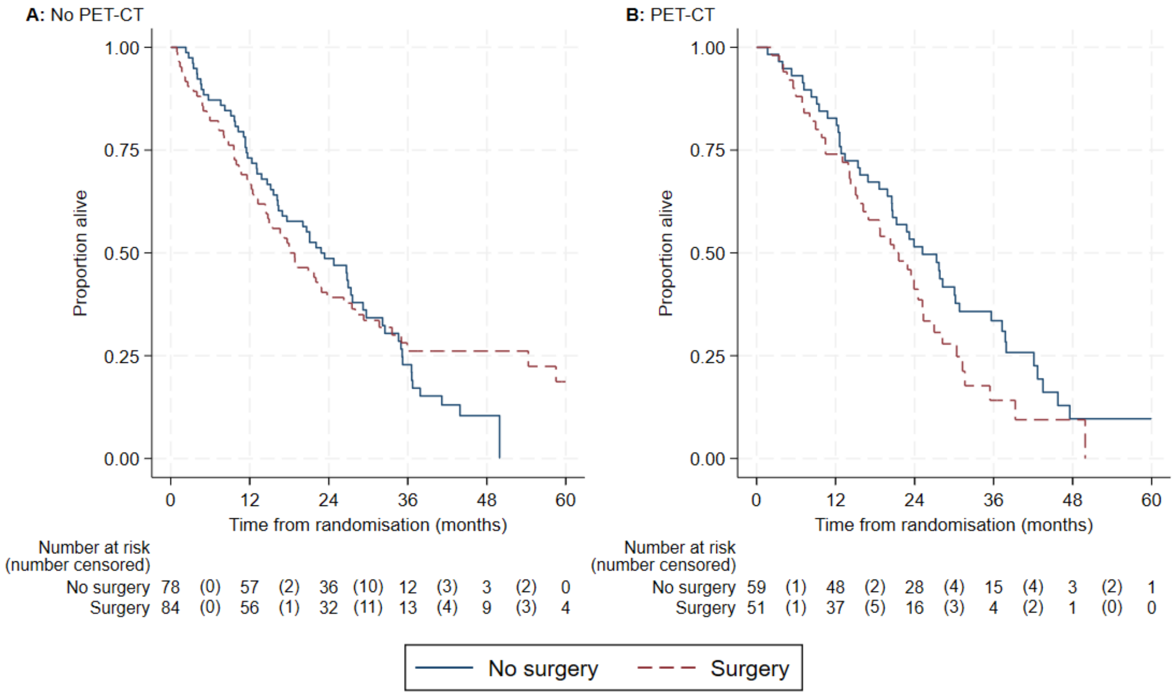

Figure S18 Overall survival by year randomised subgroups

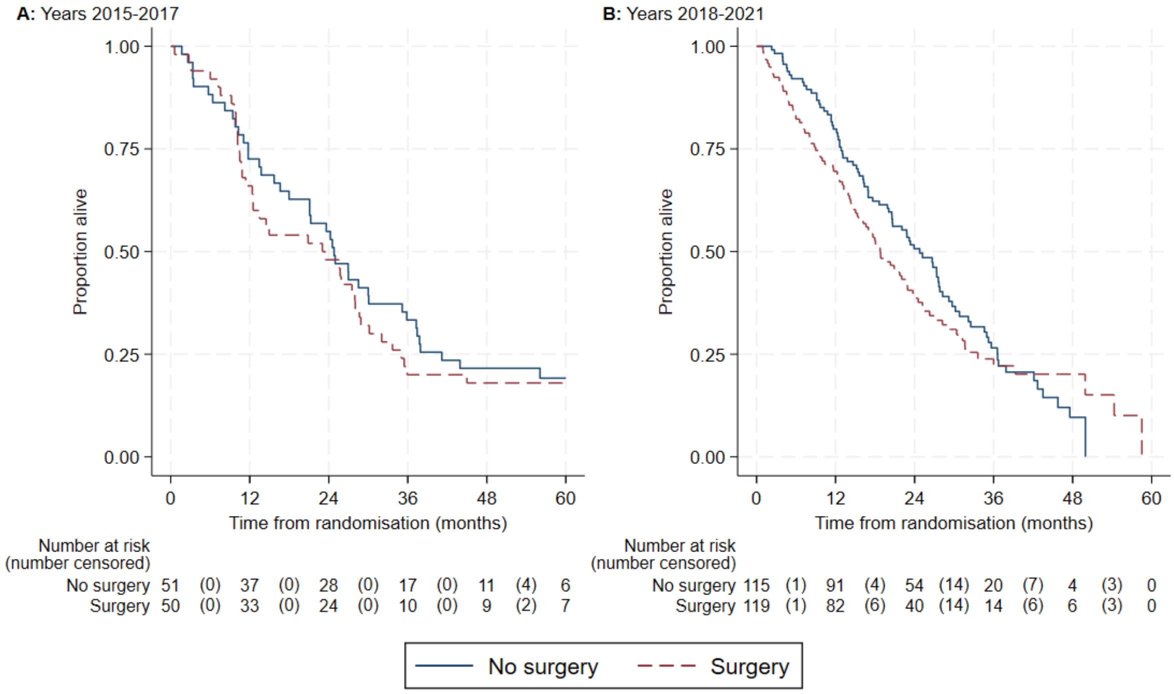

Figure S19 Overall survival for T1-2 N0 epithelioid only cohort

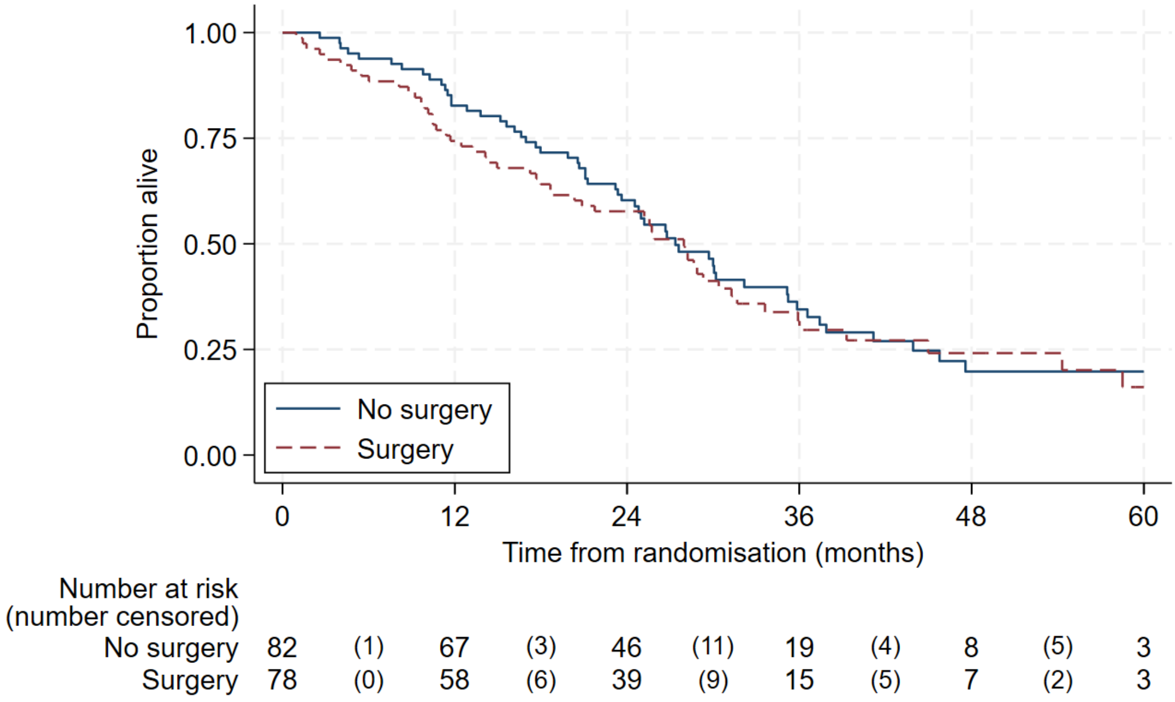

Trial Results - supplementary tables

Table S1 Baseline demography and clinical characteristics – additional information

		Randomised to surgery (n=169)	Randomised to no surgery (n=166)	Overall (n=335)
**Baseline characteristics**				
BMI (kg/m^2)		27 (24.2, 29.1)	27 (24.2, 29.4)	27 (24.2, 29.3)
Lymphocytes (x10^9/L) *		2 (0.6)	2 (0.6)	2 (0.6)
Creatinine (mmol/L) ‡		76 (66.0, 87.0)	77 (68.0, 86.5)	77 (67.0, 87.0)
Urea (mmol/L) §		5 (4.4, 6.4)	5 (4.6, 6.3)	5 (4.5, 6.3)
Neutrophils (x10^9/L) ‖		5 (4.2, 7.0)	6 (4.6, 7.0)	5 (4.4, 7.0)
**Asbestos exposure**				
Asbestos exposure	Yes	118/169 (70%)	107/166 (65%)	225/335 (67%)
	Probable	31/169 (18%)	32/166 (19%)	63/335 (19%)
	No	10/169 (6%)	7/166 (4%)	17/335 (5%)
	Unknown	10/169 (6%)	20/166 (12%)	30/335 (9%)
**Medical history**				
Respiratory comorbidity		29/169 (17%)	24/166 (15%)	53/335 (16%)
Diabetes		17/169 (10%)	16/166 (10%)	33/335 (10%)
Alcoholism		3/169 (2%)	1/166 (1%)	4/335 (1%)
Cardiovascular comorbidity		51/169 (30%)	54/166 (33%)	105/335 (31%)
Previously treated malignancy		17/169 (10%)	17/166 (10%)	34/335 (10%)
**Method of histological diagnosis**				
Thoracoscopic biopsy		112/169 (66%)	102/163 (63%)	214/332 (65%)
Core biopsy		34/169 (20%)	30/163 (18%)	64/332 (19%)
Surgical biopsy		16/169 (10%)	22/163 (14%)	38/332 (11%)
Thoracentesis		6/169 (4%)	7/163 (4%)	13/332 (4%)
Fine needle aspiration		1/169 (1%)	2/163 (1%)	3/332 (1%)
**Assessment after initial two cycles of chemotherapy**				
Evidence of disease progression but disease still surgically resectable		14/159 (9%)	14/154 (9%)	28/313 (9%)
Time from last chemotherapy to randomisation (days)		18 (15, 21)	18 (15, 21)	18 (15, 21)
Time from randomisation to start of treatment (days)		21 (16, 27)	7 (3, 10)	13 (6, 22)

Data are median (IQR), mean (SD) or n/N (%)

Missing data (surgery, no surgery): * 79 patients with missing data (40, 39), ‡ 3 patients with missing data (1. 2), § 23 patients with missing data (13, 10), ‖ 78 patients with missing data (40, 38).

Table S2 Baseline and post-op histological types pre- and post- review

Histological type pre-review	Histological type post-review	Number of patients
**Baseline histology review**		
Epithelioid mesothelioma	Epithelioid mesothelioma	34
Unable to classify	Epithelioid mesothelioma	7
Biphasic mesothelioma	Biphasic mesothelioma	7
Epithelioid mesothelioma	Biphasic mesothelioma	6
Sarcomatoid mesothelioma	Sarcomatoid mesothelioma	3
Unable to classify	Biphasic mesothelioma	2
Sarcomatoid mesothelioma	Biphasic mesothelioma	1
Epithelioid mesothelioma	Sarcomatoid mesothelioma	1
Biphasic mesothelioma	Epithelioid mesothelioma	1
Unable to classify	Sarcomatoid mesothelioma	1
**Post-op histology review**		
Biphasic mesothelioma	Biphasic mesothelioma	16
Epithelioid mesothelioma	Epithelioid mesothelioma	12
Desmoplastic mesothelioma	Desmoplastic mesothelioma	1
Desmoplastic mesothelioma	Unable to classify	1
Biphasic mesothelioma	Sarcomatoid mesothelioma	1
Sarcomatoid mesothelioma	Biphasic mesothelioma	1
Biphasic mesothelioma	Desmoplastic mesothelioma	1

Two participants with baseline histology deemed unable to classify were unable to be included in the review as histology slides could not be found.

Table S3 Reasons why screened patients were not randomised

Exclusion reason	Excluded (n=695)
**Ineligible**	**328**
Age <16 years of age	0
Not tissue confirmed epithelioid, sarcomatoid or biphasic mesothelioma	16
Disease not confined to one hemi-thorax based on CT assessment	80
Disease not deemed surgically resectable	176
Not fit for surgery	188
No capacity to provide written informed consent to participate in the trial	10
Severe shortness of breath	122
Serious concomitant disorder that would compromise participant safety during surgery	24
Liver failure	9
End stage kidney failure requiring dialysis	9
Severe heart failure	10
Prisoner	10
Patient lacks capacity to consent	9
**Not approached for consent**	**183**
Not sent PIL	90
Not interested	70
Ineligible	1
Other	22
**Did not consent**	**74**
Not interested	36
Did not feel they would benefit	8
Person reasons	3
No reason given	3
Other	24
**Other reasons**	**110**
Did not undergo two cycles of chemotherapy and have repeat CT	36
Did not start cycle 1	12/36
Disease progression	4
Not well enough	3
Patient withdrew	3
Ineligible	1
Reason not given	1
Did not start cycle 2	17/36
Could not tolerate chemotherapy	6
Patient withdrew	4
Not well enough	4
COVID-19 pandemic	2
Patient died	1
Did not have a repeat CT scan	7/36
COVID-19 pandemic	3
Patient withdrew	2
Chemotherapy not effective	1
Patient died	1
Ineligible post-consent	57
Disease progression beyond surgically resectable limits*	33
Other reason	24
Patient withdrew consent	13
COVID-19 paused trial	3
Patient died	1

Data are number of patients. * an additional two patients had disease progression beyond surgically resectable limits and did not complete the initial two cycles of chemotherapy

Table S4 Protocol deviations

	Randomised to surgery (n=169)	Randomised to no surgery (n=166)	Overall (n=335)
Any protocol deviation	13/169 (8%)	2/165 (1%)	15/334 (5%)
Participant ineligible but treated in the study	0/169 (0%)	0/166 (0%)	0/335 (0%)
Participant did not receive allocated treatment	13/169 (8%)	2/166 (1%)	15/335 (5%)
Participants' disease confirmed to not be mesothelioma	0/169 (0%)	0/165 (0%)	0/334 (0%)

Data are n/N (%)

Table S5 Reasons why randomised participants did not start chemotherapy cycle 3

Reason	Randomised to surgery (n=68)	Randomised to no surgery (n=13)	Overall (n=81)
Disease progression/clinical deterioration	25/68 (37%)	6/13 (46%)	31
Participant died	11/68 (16%)	1/13 (8%)	12
Patient decision	8/68 (12%)	2/13 (15%)	10
Clinical decision	9/68 (13%)	1/13 (8%)	10
Could not tolerate chemotherapy	4/68 (6%)	2/13 (15%)	6
Long post-operative recovery/complications	5/68 (7%)	0/13 (0%)	5
Patient started new treatments	2/68 (4%)	1/13 (8%)	3
Other	4/68 (6%)	0/13 (0%)	4

Data are n/N (%).

Table S6 Post-randomisation withdrawals

	Randomised to surgery (n=169)	Randomised to no surgery (n=166)	Overall (n=335)
**Any post-randomisation withdrawal**	**14/169 (8%)**	**10/165 (6%)**	**24/334 (7%)**
**Timing of withdrawal**			
Post-randomisation but before surgery	8/14 (57%)	10/10 (100%)	18/24 (75%)
After surgery	6/14 (43%)		6/24 (25%)
**Reason for withdrawal**			
Patient choice	8/14 (57%)	8/10 (80%)	16/24 (67%)
Patient changed their mind about the trial	0/8 (0%)	4/8 (50%)	4/16 (25%)
Referral to another centre	2/8 (25%)	0/8 (0%)	2/16 (13%)
Patient no longer wanted surgery	3/8 (38%)	0/8 (0%)	3/16 (19%)
Patient too unwell	0/8 (0%)	3/8 (38%)	3/16 (19%)
Patient did not want to complete questionnaires	1/8 (13%)	1/8 (13%)	2/16 (13%)
Patient did not want further treatment	1/8 (13%)	0/8 (0%)	1/16 (6%)
Patient chose to have procedure privately	1/8 (13%)	0/8 (0%)	1/16 (6%)
Clinician choice	6/14 (43%)	2/10 (20%)	8/24 (33%)
Disease progression	3/6 (50%)	1/2 (50%)	4/8 (50%)
Surgery no longer appropriate	3/6 (50%)	0/2 (0%)	3/8 (38%)
Patient not coping well	0/3 (0%)	1/2 (50%)	1/5 (20%)
Aspect of study withdrawn from			
QoL questionnaires and clinical follow-up	10/14 (71%)	6/10 (60%)	16/24 (67%)
QoL questionnaires and clinical follow-up but data obtained by sites from patient notes	3/14 (21%)	3/10 (30%)	6/24 (25%)
Clinical follow-up only but data obtained from patient notes	0/14 (0%)	1/10 (10%)	1/24 (4%)
QoL questionnaires only	1/14 (7%)	0/10 (0%)	1/24 (4%)
Patient willing for routine data used by the NHS to be collected in the trial	13/13 (100%)	9/10 (90%)	22/23 (96%)

Data are n/N (%). QoL=quality of life

Table S7 Surgical details

	Received surgery (n=158*)
Surgical procedure	
Extended pleurectomy/decortication	139/157 (89%)
Pleurectomy decortication	13/157 (8%)
Partial pleurectomy	3/157 (2%)
Exploration, no pleurodesis	1/157 (1%)
Other	1/157 (1%)
Resection and reconstruction	
Diaphragm resection	130/157 (83%)
Diaphragm reconstructed	128/157 (82%)
Pericardium resection	105/157 (67%)
Pericardium reconstructed	84/157 (54%)
Chest wall resection	19/157 (12%)
cT1	7/19 (37%)
cT2	3/19 (16%)
cT3	9/19 (47%)
pT3	15/19 (79%)
pT4	4/19 (21%)
Chest wall reconstructed	9/157 (6%)
Other ipsilateral lung resection	67/157 (43%)
Wedge resection	64/67 (96%)
Bilobectomy	1/67 (2%)
Lobectomy	2/67 (3%)
Completeness of resection	
R0 (no residual tumour)	5/157 (3%)
R1 (microscopic residual tumour)	127/157 (81%)
R2 (macroscopic residual tumour)	25/157 (16%)
Pleura and chest wall	
Ipsilateral parietal pleural invasion	150/157 (96%)
Ipsilateral visceral pleural invasion	136/156 (87%)
Contralateral pleural invasion	1/156 (1%)
Brachial plexus nerve invasion	1/156 (1%)
Fissural involvement	114/156 (73%)
Chest wall/endothoracic fascia invasion	46/157 (29%)
Pleura and endothoracic fascia only	24/46 (52%)
Fascia and chest wall muscle	14/46 (30%)
Fascia, chest wall and ribs	8/46 (17%)
Focus	
Single focus	13/157 (8%)
Diffuse of multifocal	144/157 (92%)
Lung parenchymal invasion	95/156 (61%)
Mediastinal invasion	
Mediastinal fat	63/156 (40%)
Mediastinal organs	5/156 (3%)
No mediastinal invasion	88/156 (56%)
Pericardial invasion	92/156 (59%)
Heart muscle invasion	0/156 (0%)
Oesophageal invasion	2/156 (1%)
Diaphragm invasion	97/156 (62%)
Spine invasion	2/156 (1%)
Pleural effusion	27/156 (17%)
Pericardial effusion	19/156 (12%)
Histological type/subtype	
Diffuse malignant mesothelioma – epithelioid mesothelioma	117/157 (75%)
Diffuse malignant mesothelioma – biphasic mesothelioma	28/157 (18%)
Diffuse malignant mesothelioma – sarcomatoid mesothelioma	7/157 (5%)
Diffuse malignant mesothelioma – desmoplastic mesothelioma	4/157 (3%)
Unable to classify	1/157 (1%)
Pathological TNM stage	
pT	
T1	15/157 (10%)
T2	15/157 (10%)
Involvement of diaphragmatic muscle	6/15 (40%)
Extension of tumour into underlying pulmonary parenchyma	13/15 (87%)
T3	98/157 (62%)
Involvement of endothoracic fascia	27/98 (28%)
Extension into mediastinal fat	48/98 (49%)
Solitary, completely resectable focus of tumour extending into soft tissues of chest wall	21/98 (21%)
Nontransmural involvement of pericardium	66/98 (67%)
T4	
Diffuse extension or multifocal masses of tumour in the chest wall	13/29 (45%)
Direct transdiaphragmatic extension of tumour to the peritoneum	4/29 (14%)
Direct extension of tumour to the contralateral pleura	0/29 (0%)

	Received surgery (n=158*)
Direct extension of tumour to mediastinal organs	4/29 (14%)
Direct extension of tumour into the spine	1/29 (3%)
Tumour extending through to the internal surface of the pericardium or involving the myocardium	15/29 (52%)
pN	
N0	81/156 (52%)
N1	65/156 (42%)
N2	10/156 (6%)
pM	
M0	150/157 (96%)
M1	7/157 (5%)
Length of hospital stay (days) §	13 (12, 14)
In-hospital mortality	6/157 (4%)
30 day mortality	6/157 (4%)
90 day mortality	14/157 (9%)

Data are n/N (%)

* 1 patient withdrew to receive surgery privately and operative details were unable to be obtained. § in-hospital deaths censored at maximum length of stay

Table S8 Causes of death

Cause of death	Randomised to surgery (n=169)	Randomised to no surgery (n=166)
**Total deaths**	**134**	**130**
Disease progression	119	122
Pneumonia	7	3
Acute respiratory distress syndrome	2	0
Myocardial ischemia	1	1
Sepsis	1	1
Cardiac failure	1	1
Atypical chest infection	1	0
Thoracic hemorrhage	1	0
COVID-19	1	0
Unknown	0	2

Data are number of patients.

Table S9 Secondary outcomes

	Randomised to surgery (n=169)	Randomised to no surgery (n=166)	Effect (95% CI)	P value
Progression free survival (months)	10.6 (6.3, 21.6)	11.0 (5.9, 19.6)	HR=0.90 (0.72, 1.11)	0.33
Number of CTCAE grade 3+ events	1 (0, 3)	0 (0, 2)	IRR=3.6 (2.3, 5.5)	<0.0001
0	62/169 (37%)	86/166 (52%)		
1	33/169 (20%)	38/166 (23%)		
2	22/169 (13%)	17/166 (10%)		
3	21/169 (12%)	12/166 (7%)		
4+	31/169 (18%)	14/166 (8%)		

Data are median (interquartile range) or n/N (%). CI=confidence interval, HR=hazard ratio, IRR=incidence rate ratio

Table S10 Safety outcomes treatment effects

	Effect (95% CI)	p value	p value adjusted for multiplicity*
Number of CTCAE grade 3+ events	IRR=3.55 (2.31, 5.45)	<0.0001	<0.0001
Any CTCAE grade 3+ event within each MedDRA SOC			
Any blood or lymphatic system disorder	IRR=0.69 (0.22, 2.16)	0.53	0.69
Any cardiac disorder	IRR=2.73 (1.11, 6.67)	0.028	0.056
Any gastrointestinal disorder	IRR=0.92 (0.45, 1.89)	0.83	0.83
Any general disorder	IRR=1.28 (0.50, 3.24)	0.61	0.69
Any infection or infestation	IRR=1.99 (1.33, 2.99)	0.0009	0.0024
Any respiratory, thoracic or mediastinal disorder	IRR=2.40 (1.52, 3.80)	0.0002	0.0007
Any surgical or medical procedure	IRR=2.23 (1.04, 4.78)	0.039	0.063
Number of CTCAE grade 3+ events using randomised group as an instrumental variable	IRR=4.02 (2.51, 6.44)	<0.0001	<0.0001
Any CTCAE grade 3+ event within each MedDRA SOC using randomised group as an instrumental variable			
Any blood or lymphatic system disorder	IRR=0.67 (0.19, 2.33)	0.53	0.69
Any cardiac disorder	IRR=3.01 (1.13, 8.02)	0.028	0.056
Any gastrointestinal disorder	IRR=0.92 (0.42, 2.02)	0.83	0.83
Any general disorder	IRR=1.31 (0.47, 3.64)	0.61	0.69
Any infection or infestation	IRR=2.13 (1.36, 3.33)	0.0009	0.0024
Any respiratory, thoracic, or mediastinal disorder	IRR=2.62 (1.58, 4.33)	0.0002	0.0007
Any surgical or medical procedure	IRR=2.41 (1.04, 5.57)	0.039	0.063

CI=confidence interval, IRR=incidence rate ratio. * Safety p-values adjusted for multiplicity using the Benjamini–Hochberg method.

Table S11 Adverse events

	Randomised to surgery (n=169)		Randomised to no surgery (n=166)	
	All events	CTCAE grade 3+	All events	CTCAE grade 3+
**Total events**	**697/162 (96%)***	**318/107 (63%)†**	**448/156 (94%)‡**	**169/80 (48%)§**
Blood and lymphatic system disorders	26/20 (12%)	9/6 (4%)	16/12 (7%)	12/10 (6%)
Anaemia	15/15 (9%)	1/1 (1%)	7/6 (4%)¶	4/4 (2%)¶
Myelosuppression	1/1 (1%)	1/1 (1%)	0/0 (-)	0/0 (-)
Neutropenia	8/6 (4%)	6/4 (2%)	9/7 (4%)	8/6 (4%)
Thrombocytopenia	2/2 (1%)	1/1 (1%)	0/0 (-)	0/0 (-)
Cardiac disorders	61/51 (30%)	30/26 (15%)	12/11 (7%)	12/11 (7%)
Angina pectoris	0/0 (-)	0/0 (-)	1/1 (1%)	1/1 (1%)
Atrial fibrillation	48/44 (26%)	19/17 (10%)	0/0 (-)	0/0 (-)
Cardiac failure	1/1 (1%)	1/1 (1%)	2/2 (1%)	2/2 (1%)
Myocardial infarction	4/4 (2%)	3/3 (2%)	1/1 (1%)	1/1 (1%)
Myocardial ischemia	1/1 (1%)	1/1 (1%)	1/1 (1%)	1/1 (1%)
Pericardial effusion	4/3 (2%)	4/3 (2%)	4/3 (2%)	4/3 (2%)
Pericarditis	0/0 (-)	0/0 (-)	2/2 (1%)	2/2 (1%)
Pulmonary oedema	1/1 (1%)	0/0 (-)	0/0 (-)	0/0 (-)
Supraventricular tachycardia	1/1 (1%)	1/1 (1%)	0/0 (-)	0/0 (-)
Tachycardia	0/0 (-)	0/0 (-)	1/1 (1%)	1/1 (1%)
Ventricular fibrillation	1/1 (1%)	1/1 (1%)	0/0 (-)	0/0 (-)
Gastrointestinal disorders	25/20 (12%)	14/13 (8%)	26/20 (12%)	22/16 (10%)
Abdominal distension	2/2 (1%)	2/2 (1%)	0/0 (-)	0/0 (-)
Abdominal hernia	2/2 (1%)	2/2 (1%)	0/0 (-)	0/0 (-)
Abdominal pain	0/0 (-)	0/0 (-)	3/3 (2%)	3/3 (2%)
Acute abdomen	0/0 (-)	0/0 (-)	1/1 (1%)	1/1 (1%)
Ascites	2/2 (1%)	1/1 (1%)	1/1 (1%)	1/1 (1%)
Colitis	1/1 (1%)	0/0 (-)	0/0 (-)	0/0 (-)
Constipation	3/3 (2%)	0/0 (-)	4/4 (2%)†	2/2 (1%)†
Diarrhoea	2/2 (1%)	2/2 (1%)	5/4 (2%)	4/3 (2%)
Dysphagia	0/0 (-)	0/0 (-)	1/1 (1%)	0/0 (-)
Intestinal perforation	1/1 (1%)	1/1 (1%)	1/1 (1%)	1/1 (1%)
Nausea	5/4 (2%)	1/1 (1%)	6/6 (4%)	6/6 (4%)
Oesophageal obstruction	0/0 (-)	0/0 (-)	1/1 (1%)	1/1 (1%)
Oesophageal perforation	1/1 (1%)	1/1 (1%)	0/0 (-)	0/0 (-)
Pancreatitis	1/1 (1%)	1/1 (1%)	0/0 (-)	0/0 (-)
Peptic ulcer/gastrointestinal haemorrhage/perforation	1/1 (1%)	1/1 (1%)	0/0 (-)	0/0 (-)
Small intestinal obstruction	1/1 (1%)	1/1 (1%)	0/0 (-)	0/0 (-)
Vomiting	3/3 (2%)	1/1 (1%)	3/2 (1%)	3/2 (1%)

	Randomised to surgery (n=169)		Randomised to no surgery (n=166)	
	All events	CTCAE grade 3+	All events	CTCAE grade 3+
General disorders and administration site conditions	257/141 (83%)	15/13 (8%)	264/150 (90%)	16/12 (7%)
Abdominal pain	3/1 (1%)	2/1 (1%)	0/0 (-)	0/0 (-)
Adverse drug reaction	0/0 (-)	0/0 (-)	1/1 (1%)	1/1 (1%)
Chest pain	3/3 (2%)	2/2 (1%)	4/4 (2%)	2/2 (1%)
Death - unknown cause	0/0 (-)	0/0 (-)	2/2 (1%)	2/2 (1%)
Disease progression	220/136 (81%)‖	-	243/147 (89%)**	-
Fatigue	1/1 (1%)	1/1 (1%)	1/1 (1%)	1/1 (1%)
Multiple organ dysfunction syndrome	2/2 (1%)	2/2 (1%)	0/0 (-)	0/0 (-)
New primary or secondary cancer	0/0 (-)	-	2/2 (1%)	-
Oedema	0/0 (-)	0/0 (-)	1/1 (1%)	1/1 (1%)
Oedema peripheral	2/2 (1%)	2/2 (1%)	1/1 (1%)	1/1 (1%)
Pain	24/22 (13%)	5/4 (2%)	5/4 (2%)†	4/3 (2%)
Perforation	1/1 (1%)	1/1 (1%)	0/0 (-)	0/0 (-)
Peripheral swelling	1/1 (1%)	0/0 (-)	1/1 (1%)	1/1 (1%)
Pyrexia	0/0 (-)	0/0 (-)	3/3 (2%)	3/3 (2%)
Infections and infestations	140/69 (41%)	124/64 (38%)	60/42 (25%)	53/39 (24%)
Atypical chest infection	1/1 (1%)	1/1 (1%)	0/0 (-)	0/0 (-)
Biliary sepsis	1/1 (1%)	1/1 (1%)	0/0 (-)	0/0 (-)
COVID-19	3/3 (2%)	3/3 (2%)	1/1 (1%)	1/1 (1%)
COVID-19 pneumonia	0/0 (-)	0/0 (-)	1/1 (1%)	1/1 (1%)
Cellulitis	1/1 (1%)	1/1 (1%)	0/0 (-)	0/0 (-)
Cystitis	0/0 (-)	0/0 (-)	1/1 (1%)	1/1 (1%)
Empyema	17/15 (9%)	15/13 (8%)	1/1 (1%)	1/1 (1%)
Infection	12/11 (7%)†	9/9 (5%)	32/23 (14%)	27/20 (12%)
Lower respiratory tract infection	6/6 (4%)	5/5 (3%)	5/5 (3%)	4/4 (2%)
Neutropenic sepsis	1/1 (1%)	1/1 (1%)	2/2 (1%)	2/2 (1%)
Pneumonia	22/22 (13%)	22/22 (13%)	12/12 (7%)	12/12 (7%)
Pneumonia/Lower respiratory tract infection	54/36 (21%)	45/31 (18%)	4/2 (1%)	3/1 (1%)
Respiratory tract infection	13/7 (4%)	12/6 (4%)	0/0 (-)	0/0 (-)
Sepsis	9/9 (5%)	9/9 (5%)	2/2 (1%)	2/2 (1%)
Suspected encephalitis	0/0 (-)	0/0 (-)	1/1 (1%)	1/1 (1%)
Suspected endocarditis	0/0 (-)	0/0 (-)	1/1 (1%)	0/0 (-)
Urinary tract infection	1/1 (1%)	0/0 (-)	0/0 (-)	0/0 (-)
Wound infection	3/3 (2%)	3/3 (2%)	0/0 (-)	0/0 (-)
Injury, poisoning and procedural complications	17/15 (9%)	11/9 (5%)	2/2 (1%)	1/1 (1%)
Fall	0/0 (-)	0/0 (-)	1/1 (1%)	0/0 (-)
Laryngeal nerve injury	6/6 (4%)	1/1 (1%)	0/0 (-)	0/0 (-)
Limb injury	1/1 (1%)	1/1 (1%)	0/0 (-)	0/0 (-)
Post procedural complication [diaphragm patch disruption]	4/4 (2%)	4/4 (2%)	0/0 (-)	0/0 (-)
Post procedural haemorrhage	1/1 (1%)	1/1 (1%)	0/0 (-)	0/0 (-)
Wound dehiscence	5/4 (2%)	4/3 (2%)	1/1 (1%)†	1/1 (1%)†
Investigations	2/2 (1%)	1/1 (1%)	2/1 (1%)	2/1 (1%)
Biopsy endometrium	0/0 (-)	0/0 (-)	1/1 (1%)	1/1 (1%)
Bronchoscopy	2/2 (1%)	1/1 (1%)	0/0 (-)	0/0 (-)
Hysteroscopy	0/0 (-)	0/0 (-)	1/1 (1%)	1/1 (1%)
Metabolism and nutrition disorders	4/4 (2%)	3/3 (2%)	8/1 (1%)	1/1 (1%)
Fluid retention	1/1 (1%)	1/1 (1%)	0/0 (-)	0/0 (-)
Hyperkalaemia	1/1 (1%)	0/0 (-)	0/0 (-)	0/0 (-)
Hypocalcemia	0/0 (-)	0/0 (-)	1/1 (1%)	0/0 (-)
Hypomagnesemia	0/0 (-)	0/0 (-)	7/1 (1%)	1/1 (1%)
Hyponatremia	2/2 (1%)	2/2 (1%)	0/0 (-)	0/0 (-)
Musculoskeletal and connective tissue disorders	0/0 (-)	0/0 (-)	2/2 (1%)	2/2 (1%)
Musculoskeletal chest pain	0/0 (-)	0/0 (-)	1/1 (1%)	1/1 (1%)
Other musculoskeletal complication	0/0 (-)	0/0 (-)	1/1 (1%)	1/1 (1%)
Nervous system disorders	7/7 (4%)	5/5 (3%)	4/4 (2%)	2/2 (1%)
Bell's palsy	0/0 (-)	0/0 (-)	1/1 (1%)	0/0 (-)
Cerebrovascular accident	3/3 (2%)	2/2 (1%)	0/0 (-)	0/0 (-)
Peripheral motor neuropathy	1/1 (1%)	1/1 (1%)	0/0 (-)	0/0 (-)
Peripheral sensory neuropathy	1/1 (1%)	0/0 (-)	1/1 (1%)	1/1 (1%)
Presyncope	1/1 (1%)	1/1 (1%)	0/0 (-)	0/0 (-)
Seizure	1/1 (1%)	1/1 (1%)	1/1 (1%)	1/1 (1%)
Transient ischemic attack	0/0 (-)	0/0 (-)	1/1 (1%)†	0/0 (-)
Psychiatric disorders	2/2 (1%)	1/1 (1%)	2/2 (1%)	2/2 (1%)
Confusional state	1/1 (1%)	1/1 (1%)	1/1 (1%)	1/1 (1%)
Depression	0/0 (-)	0/0 (-)	1/1 (1%)	1/1 (1%)
Insomnia	1/1 (1%)	0/0 (-)	0/0 (-)	0/0 (-)
Renal and urinary disorders	2/2 (1%)	1/1 (1%)	1/1 (1%)	1/1 (1%)
Acute kidney injury	1/1 (1%)†	0/0 (-)	0/0 (-)	0/0 (-)

	Randomised to surgery (n=169)		Randomised to no surgery (n=166)	
	All events	CTCAE grade 3+	All events	CTCAE grade 3+
Renal failure	0/0 (-)	0/0 (-)	1/1 (1%)	1/1 (1%)
Renal impairment	1/1 (1%)	1/1 (1%)	0/0 (-)	0/0 (-)
Respiratory, thoracic and mediastinal disorders	127/89 (53%)	84/56 (33%)	39/27 (16%)	34/27 (16%)
Acute lung injury	2/2 (1%)	2/2 (1%)	0/0 (-)	0/0 (-)
Acute respiratory distress syndrome	5/4 (2%)	5/4 (2%)	0/0 (-)	0/0 (-)
Atelectasis/Pneumothorax	5/5 (3%)	2/2 (1%)	0/0 (-)	0/0 (-)
Bronchopleural fistula	1/1 (1%)	1/1 (1%)	0/0 (-)	0/0 (-)
Chylothorax	6/6 (4%)	6/6 (4%)	0/0 (-)	0/0 (-)
Dyspnoea	23/21 (12%)†	21/20 (12%)†	23/19 (11%)	21/19 (11%)
Emphysema	2/2 (1%)	2/2 (1%)	0/0 (-)	0/0 (-)
Hypoxia	1/1 (1%)	1/1 (1%)	0/0 (-)	0/0 (-)
Lower respiratory tract infection	6/6 (4%)	5/5 (3%)	5/5 (3%)	4/4 (2%)
Pleural effusion	5/5 (3%)	4/4 (2%)	5/5 (3%)	4/4 (2%)
Pneumothorax	2/2 (1%)	1/1 (1%)	3/2 (1%)	3/2 (1%)
Pulmonary air leakage	58/55 (33%)	24/22 (13%)	2/1 (1%)††	2/1 (1%)††
Pulmonary embolism	11/8 (5%)	10/7 (4%)	3/3 (2%)	2/2 (1%)
Respiratory failure	1/1 (1%)	1/1 (1%)	0/0 (-)	0/0 (-)
Thoracic haemorrhage	1/1 (1%)	1/1 (1%)	0/0 (-)	0/0 (-)
Surgical and medical procedures	15/12 (7%)	15/12 (7%)	9/7 (4%)	8/6 (4%)
IPC insertion	0/0 (-)	0/0 (-)	3/3 (2%)	2/2 (1%)
IPC maintenance	0/0 (-)	0/0 (-)	1/1 (1%)	1/1 (1%)
Laparotomy	1/1 (1%)	1/1 (1%)	0/0 (-)	0/0 (-)
New hemofiltration/dialysis	2/2 (1%)	2/2 (1%)	0/0 (-)	0/0 (-)
Pericardial excision	1/1 (1%)	1/1 (1%)	1/1 (1%)	1/1 (1%)
Pleurodesis	0/0 (-)	0/0 (-)	1/1 (1%)	1/1 (1%)
Polypectomy	0/0 (-)	0/0 (-)	1/1 (1%)	1/1 (1%)
Re-operation	10/8 (5%)	10/8 (5%)	0/0 (-)	0/0 (-)
VATS debridement	0/0 (-)	0/0 (-)	1/1 (1%)	1/1 (1%)
VATS debridement and rib resection	1/1 (1%)	1/1 (1%)	0/0 (-)	0/0 (-)
VATS decortication	0/0 (-)	0/0 (-)	1/1 (1%)	1/1 (1%)
Vascular disorders	12/12 (7%)	5/5 (3%)	1/1 (1%)	1/1 (1%)
Deep vein thrombosis	4/4 (2%)	1/1 (1%)	0/0 (-)	0/0 (-)
Embolism	0/0 (-)	0/0 (-)	1/1 (1%)	1/1 (1%)
Embolism venous	1/1 (1%)	0/0 (-)	0/0 (-)	0/0 (-)
Haemorrhage	5/5 (3%)	2/2 (1%)	0/0 (-)	0/0 (-)
Hypotension	1/1 (1%)	1/1 (1%)	0/0 (-)	0/0 (-)
Superior vena caval obstruction	1/1 (1%)	1/1 (1%)	0/0 (-)	0/0 (-)

Data are number of events/number of patients (% of patients). CTCAE grade 3+ events are a subset of total events reported. Adverse events are coded according to the Medical Dictionary for Regulatory Activities system organ class.

Events in patients who crossed over: * 23 events in 12 patients, † 1 event in 1 patient, ‡ 12 events in 2 patients, § 6 events in 2 patients, ¶ 2 events in 2 patients, ‖ 20 events in 12 patients, ** 4 events in 2 patients, †† 2 events in 1 patient.

Table S12 QLQ-C30 global health status and functional scale scores effect estimates

	Type of treatment effect estimate	Effect (95% CI)	p-value	p-value adjusted for multiplicity*
Global health status/QoL	Test for time*treatment interaction		0.13	
	Overall treatment effect	MD=-5.81 (-9.73, -1.89)	0.0038	0.013
Physical functioning	Test for time*treatment interaction		0.002	
	Treatment effect at 6 weeks	MD=-11.46 (-15.39, -7.52)	<0.0001	<0.0001
	Treatment effect at 6 months	MD=-8.92 (-12.33, -5.51)	<0.0001	<0.0001
	Treatment effect at 12 months	MD=-5.63 (-9.36, -1.91)	0.0030	0.011
	Treatment effect at 18 months	MD=-2.34 (-7.27, 2.58)	0.35	0.57
	Treatment effect at 24 months	MD=0.95 (-5.60, 7.49)	0.78	0.86
Social functioning	Test for time*treatment interaction		0.16	
	Overall treatment effect	MD=-10.87 (-16.07, -5.66)	<0.0001	0.0004
Role functioning	Test for time*treatment interaction		0.016	
	Treatment effect at 6 weeks	MD=-15.77 (-22.03, -9.50)	<0.0001	<0.0001
	Treatment effect at 6 months	MD=-13.05 (-18.61, -7.48)	<0.0001	<0.0001
	Treatment effect at 12 months	MD=-9.51 (-15.41, -3.62)	0.0016	0.0063
	Treatment effect at 18 months	MD=-5.98 (-13.39, 1.44)	0.11	0.24
	Treatment effect at 24 months	MD=-2.44 (-12.01, 7.13)	0.62	0.78
Cognitive functioning	Test for time*treatment interaction		0.86	
	Overall treatment effect	MD=-0.15 (-3.51, 3.22)	0.93	0.93
Emotional functioning	Test for time*treatment interaction		0.064	
	Treatment effect at 6 weeks	MD=-1.04 (-4.73, 2.66)	0.58	0.78
	Treatment effect at 6 months	MD=0.28 (-3.05, 3.61)	0.87	0.92
	Treatment effect at 12 months	MD=1.99 (-1.64, 5.63)	0.28	0.49
	Treatment effect at 18 months	MD=3.70 (-0.93, 8.33)	0.12	0.24
	Treatment effect at 24 months	MD=5.41 (-0.57, 11.39)	0.076	0.18

CI=confidence interval, MD=mean difference. Scores range from 0 to 100. Higher scores represent higher levels of functioning. *HRQoL p-values adjusted for multiplicity using the Benjamini–Hochberg method.

Table S13 QLQ-C30 symptom scale scores effect estimates

	Type of treatment effect	Effect (95% CI)	p-value	p-value adjusted for multiplicity*
Diarrhoea	Time*treatment interaction		0.29	
	Overall treatment effect	OR=0.79 (0.49, 1.26)	0.32	0.53
Fatigue	Time*treatment interaction		0.020	
	Treatment effect at 6 weeks	MD=10.78 (4.65, 16.92)	0.0007	0.0034
	Treatment effect at 6 months	MD=9.49 (3.88, 15.09)	0.0011	0.0048
	Treatment effect at 12 months	MD=1.02 (-5.61, 7.65)	0.77	0.86
	Treatment effect at 18 months	MD=-0.49 (-7.78, 6.80)	0.90	0.92
	Treatment effect at 24 months	MD=-4.12 (-12.98, 4.75)	0.37	0.58
Nausea	Time*treatment interaction		0.71	
	Overall treatment effect	OR=1.05 (0.63, 1.73)	0.86	0.92
Pain	Time*treatment interaction		<0.001	
	Treatment effect at 6 weeks	MD=25.98 (19.64, 32.31)	<0.0001	<0.0001
	Treatment effect at 6 months	MD=6.98 (1.44, 12.52)	0.015	0.042
	Treatment effect at 12 months	MD=6.18 (-0.66, 13.02)	0.078	0.18
	Treatment effect at 18 months	MD=4.96 (-3.18, 13.10)	0.24	0.42
	Treatment effect at 24 months	MD=1.74 (-7.50, 10.99)	0.72	0.85
Dyspnoea	Time*treatment interaction		<0.001	
	Treatment effect at 6 weeks	OR=4.28 (2.42, 7.55)	<0.0001	<0.0001
	Treatment effect at 6 months	OR=2.90 (1.75, 4.80)	<0.0001	0.0002
	Treatment effect at 12 months	OR=1.43 (0.81, 2.50)	0.22	0.40
	Treatment effect at 18 months	OR=1.38 (0.67, 2.82)	0.38	0.58
	Treatment effect at 24 months	OR=1.23 (0.53, 2.83)	0.63	0.78
Insomnia	Time*treatment interaction		0.046	
	Treatment effect at 6 weeks	OR=2.15 (1.08, 4.28)	0.028	0.073
	Treatment effect at 6 months	OR=0.81 (0.42, 1.58)	0.55	0.75
	Treatment effect at 12 months	OR=0.88 (0.41, 1.88)	0.73	0.85
	Treatment effect at 18 months	OR=0.54 (0.22, 1.29)	0.16	0.31
	Treatment effect at 24 months	OR=0.67 (0.25, 1.81)	0.43	0.61
Appetite loss	Time*treatment interaction		0.002	
	Treatment effect at 6 weeks	OR=2.93 (1.30, 6.60)	0.0084	0.027
	Treatment effect at 6 months	OR=2.70 (1.25, 5.83)	0.011	0.033
	Treatment effect at 12 months	OR=1.06 (0.43, 2.63)	0.90	0.92
	Treatment effect at 18 months	OR=2.29 (0.83, 6.32)	0.11	0.24
	Treatment effect at 24 months	OR=0.26 (0.08, 0.85)	0.024	0.062
Constipation	Time*treatment interaction		0.69	
	Overall treatment effect	OR=1.42 (0.87, 2.30)	0.16	0.31

	Type of treatment effect	Effect (95% CI)	p-value	p-value adjusted for multiplicity*
Financial difficulties	Time*treatment interaction		0.008	
	Treatment effect at 6 weeks	OR=10.61 (2.99, 37.61)	0.0001	0.0006
	Treatment effect at 6 months	OR=1.26 (0.41, 3.93)	0.67	0.80
	Treatment effect at 12 months	OR=1.75 (0.47, 6.51)	0.40	0.59
	Treatment effect at 18 months	OR=1.44 (0.31, 6.62)	0.63	0.78
	Treatment effect at 24 months	OR=0.45 (0.07, 3.01)	0.42	0.60

CI=confidence interval, OR=odds ratio, MD=mean difference. Scores range from 0 to 100. Higher scores indicate a higher level of symptoms. *HRQoL p-values adjusted for multiplicity using the Benjamini–Hochberg method.

Table S14 Other clinical trials joined during trial participation

	Randomised to surgery (n=169)	Randomised to no surgery (n=166)
**Any participation in another clinical trial**	**22/169 (13%)**	**34/166 (21%)**
CONFIRM	8	16
VIM	8	5
PROMISE-meso	3	5
FAK-PD1	2	1
MiST1	3	0
MiST5	2	1
INFINITE	0	2
MiST3	0	2
MiST4	0	2
SYSTEMS-2	2	0
Bayer thorium	1	0
Epizyme	0	1
MiST2	1	0
Oraxol food effect trial	0	1
SIMPLE	0	1

Data are n/N (%) or number of patients

Table S15 Secondary outcome sensitivity analyses

	Primary analysis		Analysis using randomised group as instrumental variable	
	Effect size (95% CI)	P value	Effect size (95% CI)	P value
Progression-free survival	HR=0.90 (0.72, 1.11)	0.33	HR=0.89 (0.70, 1.13)	0.33
Number of CTCAE 3+ events	IRR=3.55 (2.31, 5.45)	<0.0001	IRR=4.02 (2.51, 6.44)	<0.0001

CI=confidence interval, HR=hazard ratio, IRR=incidence rate ratio

Table S16 Exploratory analysis of surgical site experience level

	HR (95% CI)	p-value for three level factor
Expert surgical site vs chemotherapy alone	HR=1.18 (0.95, 1.47)	0.29
Non-expert surgical site vs chemotherapy alone	HR=1.83 (0.49, 6.85)	
Expert surgical site vs non-expert surgical site	HR=0.65 (0.17, 2.40)	

CI=confidence interval, HR=hazard ratio

Economic Evaluation Results – additional information

The table below shows the average costs and QALYs per participant in each group. Costs are statistically significantly higher in the surgery group, and QALYs are significantly lower. Surgery is clearly not cost-effective, and is said to be “dominated” by chemotherapy alone.

Table S17 Cost-effectiveness results

Cost-effectiveness element	Randomised to surgery (n=169) Mean (95% CI)	Randomised to no surgery (n=166) Mean (95% CI)	Surgery versus chemotherapy alone Mean difference (95% CI)
Total costs (£)	30,436 (27,753 to 33,119)	15,805 (13,995 to 17,614)	+14,631 (11,279 to 17,983)
Total costs (US$)	41,871 (38,180 to 45,562)	21,743 (19,253 to 24,232)	+20,128 (15,517 to 24,740)
QALYs	1.02 (0.94 to 1.10)	1.21 (1.13 to 1.29)	-0.19 (-0.30 to -0.08)

CI=confidence interval, QALY=quality-adjusted life-year

References

1. Hernández-Alava M, Pudney S. Eq5Dmap: A Command for Mapping between EQ-5D-3L and EQ-5D-5L. The Stata Journal. 2018;18(2):395-415.
2. Bristol-Myers Squibb Company. PLATINOL® (cisplatin for injection, USP) [package insert]. U.S. Food and Drug Administration website. https://www.accessdata.fda.gov/drugsatfda_docs/label/2010/018057s079lbl.pdf. Revised May 2010. Accessed July 5, 2023.
3. Bristol-Myers Squibb Company. PARAPLATIN® (carboplatin) Injection [package insert]. U.S. Food and Drug Administration website. https://www.accessdata.fda.gov/drugsatfda_docs/label/2010/020452s005lbl.pdf. Revised July 2010. Accessed July 5, 2023.
4. Zydus Hospira Oncology Private Ltd. PEMETREXED injection [package insert]. U.S. Food and Drug Administration website. https://www.accessdata.fda.gov/drugsatfda_docs/label/2022/214218Orig1s000lbl.pdf. Revised April 2022. Accessed July 5, 2023.
